# A matter of differentiation: equine enteroids as a model for the in vivo intestinal epithelium

**DOI:** 10.1186/s13567-024-01283-0

**Published:** 2024-03-16

**Authors:** Christina Windhaber, Anna Heckl, Georg Csukovich, Barbara Pratscher, Iwan Anton Burgener, Nora Biermann, Franziska Dengler

**Affiliations:** 1 Institute of Physiology, Pathophysiology and Biophysics, University of Veterinary Medicine, Vienna, Austria; 2 Division of Small Animal Internal Medicine, University of Veterinary Medicine, Vienna, Austria; 3 Clinical Unit of Equine Surgery, University of Veterinary Medicine, Vienna, Austria

**Keywords:** Organoid, jejunum, colon, horse, three-dimensional cell culture, colonoid, differentiation, stem cells, enterocytes

## Abstract

Epithelial damage due to gastrointestinal disorders frequently causes severe disease in horses. To study the underlying pathophysiological processes, we aimed to establish equine jejunum and colon enteroids (eqJE, eqCE) mimicking the in vivo epithelium. Therefore, enteroids were cultivated in four different media for differentiation and subsequently characterized histomorphologically, on mRNA and on protein level in comparison to the native epithelium of the same donor horses to identify ideal culture conditions for an in vitro model system. With increasing enterocyte differentiation, the enteroids showed a reduced growth rate as well as a predominantly spherical morphology and less budding compared to enteroids in proliferation medium. Combined or individual withdrawal of stem cell niche pathway components resulted in lower mRNA expression levels of stem cell markers and concomitant differentiation of enterocytes, goblet cells and enteroendocrine cells. For eqCE, withdrawal of Wnt alone was sufficient for the generation of differentiated enterocytes with a close resemblance to the in vivo epithelium. Combined removal of Wnt, R-spondin and Noggin and the addition of DAPT stimulated differentiation of eqJE at a similar level as the in vivo epithelium, particularly with regard to enterocytes. In summary, we successfully defined a medium composition that promotes the formation of eqJE and eqCE consisting of multiple cell types and resembling the in vivo epithelium. Our findings emphasize the importance of adapting culture conditions to the respective species and the intestinal segment. This in vitro model will be used to investigate the pathological mechanisms underlying equine gastrointestinal disorders in future studies.

**Supplementary Information:**

The online version contains supplementary material available at 10.1186/s13567-024-01283-0.

## Introduction

Gastrointestinal disorders referred to as colic are a major cause of morbidity and mortality in horses [1, 2]. It is caused by different underlying pathologies, with displacement and strangulation of the small or large intestine, i.e., jejunum or colon, being the most commonly diagnosed [3]. Independent of the underlying disease, a major pathophysiological factor is the disruption of the intestinal epithelial integrity and subsequent compromised barrier function [4–6]. The intestinal epithelium is a highly dynamic tissue, whose main functions are the protection of the body from luminal pathogens and the uptake of nutrients by vectorial transport [7]. Since the absorptive and protective function of the intestine is based on an intact and functional epithelium [8], a better understanding of the pathophysiological processes leading to epithelial damage and mechanisms of epithelial repair and adaptation is crucial for improving the therapeutic options to treat equine colic.

Currently, research in this field is conducted either in vivo using patient material or animal experiments or in vitro with intestinal epithelial cell lines [9, 10]. Both approaches are not ideal to understand the epithelial pathophysiology. Investigations in vivo are complex and may either be confounded by the rather heterogenous horse population or, when using rodent models instead, may not be translatable to the equine patient. Additionally, ethical concerns must be considered, and cell level in vitro experiments offer better insights into the pathophysiological processes on the epithelial level without being biased by systemic influences. However, most cell lines used for research are tumour-derived or otherwise immortalized clones of individual cells and may not represent the original tissue and its pathophysiology anymore. Intestinal epithelial cell lines in particular have weaknesses, as they show reduced complexity and low physiological relevance, whereas the in vivo epithelium consists of more than one cell type [10]. Besides, at this point no equine intestinal epithelial cell line is available and although there have been attempts to culture primary epithelial cells, a major limitation is their short life span [11–13]. Consequently, there is a dire need for an in vitro model which mimics species-specific in vivo conditions ideally suitable for long term studies.

Since intestinal stem cells (ISC) have the ability of self-renewal and the potential to generate all differentiated mature cell types of the epithelium (enterocytes, goblet cells, enteroendocrine cells and Paneth cells) [8, 14, 15], they are increasingly used to develop intestinal epithelial enteroids. These three-dimensional models can be cultured long-term without loss of epithelial characteristics [16] displaying genetic stability [17] and can be employed to investigate epithelial pathophysiology. The isolated ISC are embedded in a laminin rich extracellular matrix, where they self-organize and form a three-dimensional multilobular structure with a pseudolumen [15, 16]. The culture media usually contain several factors that play a crucial role in activation and control of the stem cell niche and enhance cell proliferation [17–22]. At the same time increased proliferation also means less differentiation and thus a lack of similarity to the in vivo epithelium. Withdrawal or inhibition of these growth-promoting factors induces the differentiation of the cells into the mature cell types [23–25]. Most studies, however, have not compared the grade of differentiation of the enteroids with the in vivo tissue but with undifferentiated enteroids only [22, 25–28], making it impossible to judge the potential of the model to replace in vivo experiments. Furthermore, the conditions for differentiation are not identical across species and need further refinement [26]. It has been demonstrated that differentiation of specific cell types can be promoted by modulation of the medium composition [29]. Additionally, bioengineering research is being conducted to induce more in vivo-like structures [30]. Human [23, 26, 31, 32], murine [26, 33], canine [34] as well as farm animal [35–43] derived enteroid models are well established. However, to date there are only few studies on small intestinal equine enteroids [44–47], and none dealing with equine colon enteroids, and a quantitative comparison with the original equine intestinal epithelium of the donors is lacking. Nevertheless, this is a prerequisite for using enteroids as a true alternative to animal experiments with potential for translation to the in vivo situation. Therefore, we aimed to characterize equine jejunum enteroids (eqJE) and equine colon enteroids (eqCE) grown under various conditions regarding their cellular composition and degree of differentiation in comparison to the original epithelium concentrating on enterocytes as the predominant cell type.

## Materials and methods

### Isolation and cultivation of eqJE and eqCE

The enteroids were generated from tissue specimens of horses euthanized at the equine clinic of the University of Veterinary Medicine, Vienna for unrelated reasons with informed consent of the owners. All donor horses were intestinally healthy. For the generation of eqJE, we harvested distal jejunum from a total of *N* = 9 horses (6 mares, 2 geldings, 1 stallion) of various breeds with an age between 7 months and 25 years (mean = 12.2 ± 7.27 years). For eqCE, large colon, precisely pelvic flexure, of *N* = 8 horses (5 mares, 3 geldings) of various breeds aged between 4 and 20 years (mean = 11 ± 4.92 years) was sampled. Within 30 min after humane euthanasia of the horses, a 20–30 cm long section of intestine was excised, opened longitudinally, and rinsed with 4 °C 0.9% sterile NaCl until the solution remained clear. Then the epithelium was separated from the underlying muscle layer by scraping using a scalpel. Thus, the isolated epithelium contained minor remnants of underlying connective tissue. The pieces were placed in ice-cold transport solution for approximately 45 min. The transport solution consisted of Dulbecco’s Phosphate Buffered Saline (DPBS, Merck, Vienna, Austria) supplemented with 0.5 mg/mL Gentamicin (Merck, Vienna, Austria) and 100 U/mL penicillin/streptomycin (Merck, Vienna, Austria). Additionally, pieces of the same isolated epithelium from the same horse were stored immediately at −80 °C and in RNAlater^®^ (Merck, Vienna, Austria). Full thickness samples were fixed with formaldehyde (ROTI^®^ Histofix 4%, Carl Roth GmbH + Co. KG, Karlsruhe, Germany) for subsequent histological comparison with the enteroids.

**Figure 1:**
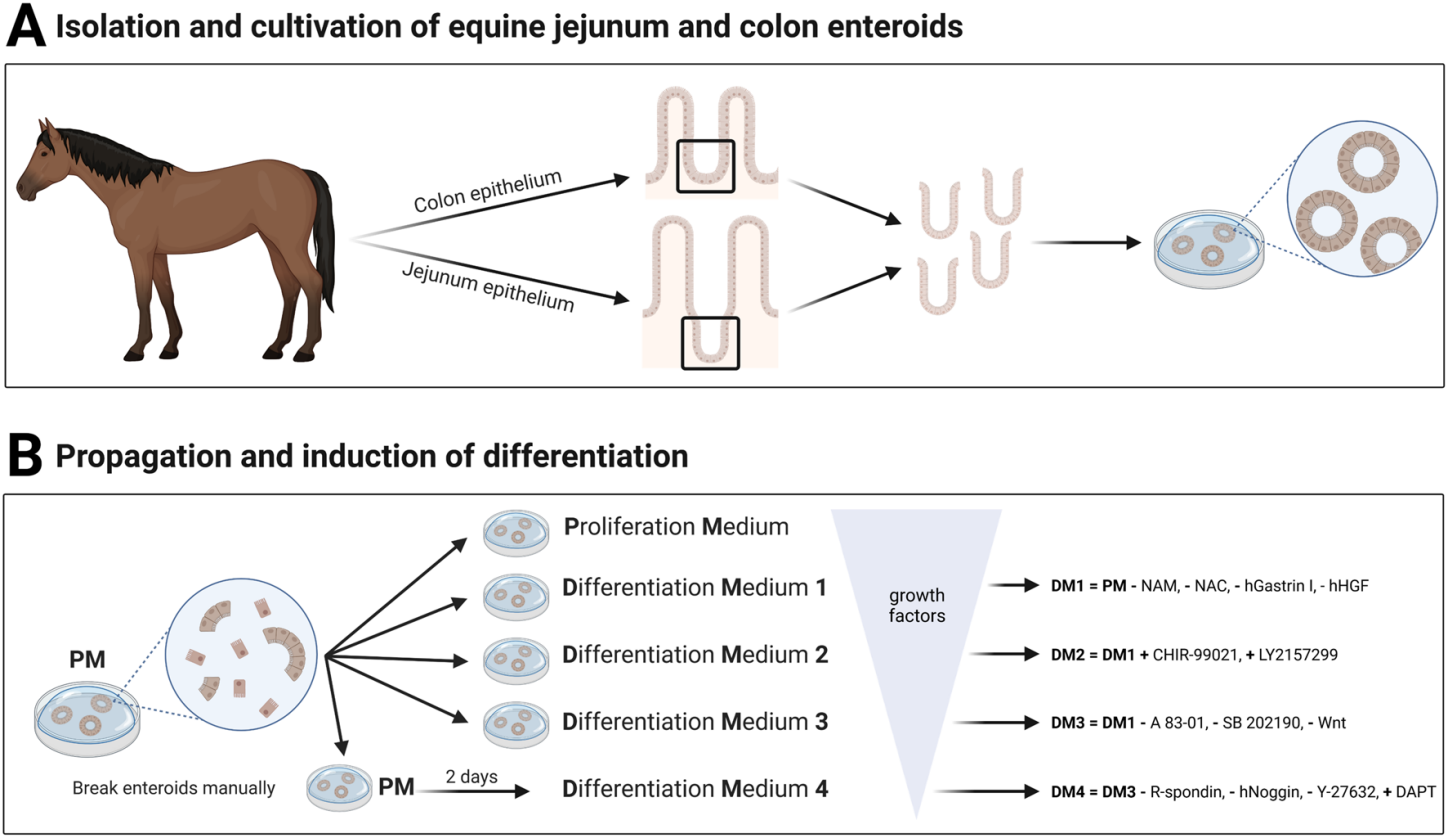
**Schematic illustration of the isolation of intestinal crypts and cultivation of eqJE and eqCE (A) and the cultivation of eqJE and eqCE with differently composed media (B).** NAM: Nicotinamide, NAC: N-Acetyl-L-Cysteine, hHGF: human hepatocyte growth factor, Wnt: Wnt3A conditioned medium, R-spondin: Rspo1 conditioned medium, Y-27632: Y-27632 dihydrochloride. Detailed media compositions are listed in Table 1. Created with BioRender.

For crypt isolation, we used a previously published protocol with some modifications [48]. Briefly, the epithelium was cut into small pieces and placed in a solution of DPBS and 5 mM ethylenediaminetetraacetic acid (EDTA, pluriSelect Life Science, Leipzig, Germany). To dissociate the crypts from the tissue, the pieces were vigorously pipetted before incubation on a benchtop roller at 4 °C. After gravity-induced sedimentation, the supernatant was discarded, and the process was repeated at least three times. After the third repetition, the supernatant (“fraction III”) was collected in a tube and examined under the microscope. Accordingly, fractions IV-VI were generated and the fraction with the highest purity of intestinal crypts was chosen for seeding. Therefore, the selected fraction was centrifuged at 80 *g* for 5 min at 4 °C. Thereafter, the pellet was washed with basal medium (BM, see Table 1) and was centrifuged again. Afterwards, the crypts were mixed with extracellular matrix components (Geltrex^™^, Thermo Fisher Scientific, Waltham, MA, USA), seeded onto a 24 well plate (Biologix^®^, Hallbergmoos, Germany), covered with proliferation medium (PM, Table 1) and incubated in a humidified atmosphere at 37 °C with 5% CO_2_. A schematic illustration of the isolation and initial cultivation process is shown in Figure 1A.

**Table 1:** **Medium compositions.**

Component	PM	DM1	DM2	DM3	DM4	Manufacturer; # Catalog Number
Basal medium (BM)^*^	+	+	+	+	+	
B-27^™^ supplement	1 ×	1 ×	1 ×	1 ×	1 ×	Thermo Fisher Scientific, Waltham, MA, USA; # 17504001
N-2	1 ×	1 ×	1 ×	1 ×	1 ×	Thermo Fisher Scientific, Waltham, MA, USA; # 17502001
Nicotinamide	10 mM	−	−	−	−	Merck, Vienna, Austria; # 72 340
n-Acetyl-l-Cysteine	1 mM	−	−	−	−	Merck, Vienna, Austria; # A9165
A 83–01	500 nM	500 nM	500 nM	−	−	Hycultec GmbH, Beutelsbach, Germany; # HY-10432
SB 202190	10 µM	10 µM	10 µM	−	−	Hycultec GmbH, Beutelsbach, Germany; # HY-10295
hGastrin I	10 nM	−	−	−	−	Hycultec GmbH, Beutelsbach, Germany; # HY-P1097
hHGF	50 ng/mL	−	−	−	−	Thermo Fisher Scientific, Waltham, MA, USA; # 100-39H
mEGF	50 ng/mL	50 ng/mL	50 ng/mL	50 ng/mL	50 ng/mL	Thermo Fisher Scientific, Waltham, MA, USA; # 315–09
CHIR-99021	−	−	2.5 µM	−	−	BioGems; Westlake Village, CA, USA; # 2 520 691
LY2157299	−	−	500 nM	−	−	TargetMol^®^, Wellesley Hills, MA, USA; # T2510
hNoggin	100 ng/mL	100 ng/mL	100 ng/mL	100 ng/mL	−	Thermo Fisher Scientific, Waltham, MA, USA; # 120-10C
Wnt3A conditioned medium^#^	43% v/v	43% v/v	43% v/v	−	−	In house manufacturing (Division of Small Animal Internal Medicine, University of Veterinary Medicine Vienna)
Rspo1 conditioned medium^﻿#^	10% v/v	10% v/v	10% v/v	10% v/v	−	In house manufacturing (Division of Small Animal Internal Medicine, University of Veterinary Medicine Vienna)
Gentamicin	20 µg/mL	20 µg/mL	20 µg/mL	20 µg/mL	20 µg/mL	Merck, Vienna, Austria; # G1272
DAPT	−	−	−	−	5 µM	Hycultec GmbH, Beutelsbach, Germany; # HY-13027
Y-27632 dihydrochloride	10 µM	10 µM	10 µM	10 µM	−	TargetMol^®^, Wellesley Hills, MA, USA; # T1725

*BM consists of Advanced DMEM/F12 (Thermo Fisher Scientific, Waltham, MA, USA), 10 mM HEPES (Merck, Vienna, Austria), 2 mM GlutaMAX^™^ (Thermo Fisher Scientific, Waltham, MA, USA), 100 U/mL penicillin/streptomycin (Merck, Vienna, Austria). Final concentrations are given.

^﻿#^Cells for conditioned medium production were provided by Bio-Techne^®^, Abingdon, UK (Cultrex^®^ HA-R-Spondin1-Fc 293 T cells) and as a generous gift from Prof. Dr. Clevers (L-Wnt-3A cells).

### Propagation and cryo-preservation of equine enteroids

The enteroids were passaged once to twice a week in a ratio between 1:2 and 1:3, depending on the amount and size of enteroids. So far it was possible to maintain the enteroids for at least 20 passages without morphological changes. Therefore, the Geltrex^™^ domes were mechanically dissolved and the enteroids were further disrupted with a 27 G needle. After centrifugation at 240 *g* for 5 min at 4 °C the fragments were seeded on a 24 well plate as described above. For short time storage at −80 °C, the disrupted enteroids were resuspended in Recovery^™^ Cell Culture Freezing Medium (Thermo Fisher Scientific, Waltham, USA) supplemented with 10 µM Y-27632 dihydrochloride (TargetMol^®^, Wellesley Hills, MA, USA).

### Induction of differentiation

To induce a differentiation of epithelial cells, enteroids of passage four or higher were cultivated with either PM or one of four differentiation media (DM1-4, Table 1). The media compositions were designed based on previously published papers [29, 34, 42, 44] and are detailed in Table 1. As cultivation of newly split enteroids with DM4 led to reduced growth and cell death after a few days, enteroids were cultivated for two days in PM before changing to DM4, whereas all other media were applied directly after reseeding as illustrated in Figure 1B. The medium was changed every 2–3 days and the growth and morphology of the enteroids were monitored daily. Sampling for downstream analyses took place when the enteroids reached a size that required splitting. Therefore, eqJE were sampled after five days and eqCE after four days in culture.

### Microscopy and growth measurements

To estimate the expansion and growth rate of the enteroids, images were taken with a brightfield Zeiss Axio Vert.A1 and an Axiocam ERs 5 s Zeiss camera (Carl Zeiss Microscopy GmbH, Oberkochen, Germany). Circumference measurements of enteroids were used to determine the growth rate as previously described [49–51]. At the beginning and after four days of cultivation, the circumference of the enteroids was measured using the freehand selection tool in Fiji ImageJ64 [52]. For each biological replicate 7–12 enteroids were measured and the mean values at day one of cultivation were used as individual calibrator (= 100%) for each biological replicate. The growth of the corresponding enteroids at day four was calculated relative to that.

### Stainings and imaging

For histologic evaluation, enteroids from one well of a 24 well plate were harvested and fixed with 2% *v/v* formaldehyde (ROTI^®^ Histofix 4%, Carl Roth GmbH + Co. KG, Karlsruhe, Germany) for 20 min at room temperature. Native jejunum and colon samples of the same horses were fixed with 4% *v/v* formaldehyde for 24 h and stored in 70% ethanol at 4 °C. Formalin-fixed cryosections (6 µm) of enteroids and the corresponding tissue samples were stained using haematoxylin and eosin (H&E) as well as Periodic acid-Schiff (PAS).

Additionally, immunohistochemical (IHC) stainings of chromogranin A (CHGA) as a marker for enteroendocrine cells were performed with paraffin-embedded sections (2.5 µm). Therefore, the samples were blocked with 1.5% goat serum (Merck, Vienna, Austria) diluted in PBS, incubated over night at 4 °C with primary antibody (Anti-Chromogranin A, rabbit polyclonal IgG antibody, Abcam, Cambridge, UK; #ab45179; 1:20 000), for 30 min with secondary antibody (Goat Anti-Rabbit HRP, Medac GmbH, Wedel, Germany; #DPVR110HRP) according to the manufacturer’s instructions and counterstained with haematoxylin acidic. Pictures were taken with a Zeiss Axiocam 503 color (Carl Zeiss Microscopy GmbH, Oberkochen, Germany).

### mRNA extraction and two-step RT-qPCR

For extraction of total RNA, one 24 well with enteroids was harvested at the end of the cultivation period, washed twice with DPBS, spun down at 240 g for 5 min at 4 °C, mixed with lysis buffer from the ReliaPrep^™^ RNA Tissue Miniprep System (Promega GmbH, Walldorf, Germany) and stored at −20 °C. Similarly, 30 mg of native epithelium were mixed with lysis buffer and homogenised using a mixer mill (Retsch MM200, Merck, Vienna, Austria) for 5 min with 3 Hz. RNA extraction, cDNA synthesis and qPCR analysis were performed according to the manufacturers’ protocols as previously published [53] and according to the MIQE guidelines [54]. RNA concentration and quality were determined using a spectrophotometer (DS-11, DeNovix, Wilmington, USA). Additional random sampling confirmed an average RNA integrity number (RIN) of 9.5 (range 7.2–10.0). Individual annealing temperatures for each gene are given in Additional file 1. To minimize inaccuracies, all samples were run in duplicates. The deviation of the C_t_ values of the technical replicates was ≤ 0.7; if it was higher, the data were discarded, and the run was repeated. The ΔΔC_t_ method was used to compare the mRNA expression. Normalization of the samples was achieved using the same amount of RNA for processing and normalizing the target genes’ C_t_ to the geometric means of the C_t_ values of the reference genes peptidylprolyl isomerase A (*PPIA*), ribosomal protein L32 (*RPL32*) and hypoxanthine phosphoribosyltransferase 1 (*HPRT1*) for jejunum and *PPIA*, *HPRT1* and ribosomal protein L4 (*RPL4*) for colon epithelium, respectively. The reference genes’ stability was checked with the program RefFinder [55].

We measured the mRNA expression levels of marker genes for ISC (olfactomedin 4, *OLFM4*), Paneth cells (lysozyme, *LYZ*), goblet cells (mucin 2, *MUC2*), enteroendocrine cells (*CHGA*) and the most abundant cell type in the small intestine, enterocytes. According to their pivotal function in the uptake of nutrients and barrier formation, we assessed the expression of the transport proteins Na^+^/K^+^-ATPase subunit alpha-1 (*Na*^+^*/K*^+^*-ATPase*), cystic fibrosis transmembrane conductance regulator (*CFTR*), peptide transporter 1 (*PepT1*), excitatory amino acid transporter 3 (*EAAT3*), amino acid transporter B0 (*ATB0*), glucose transporter 1 (*GLUT1*) and sodium/glucose-cotransporter 1 (*SGLT1*), of the tight junction proteins occludin (*OCLN*), claudin 4 (*CLDN4*), claudin 7 (*CLDN7*) and claudin 12 (*CLDN12*), the cell adhesion proteins cadherin 1 (*CDH1*) and epithelial cell adhesion molecule (*EPCAM*) and of villin (*VIL1*), which is an important part of the brush border membrane. Primers used for qPCR are listed in Additional file 1.

### Protein expression

For total protein extraction the enteroids were harvested as described above for mRNA extraction and the pellet was stored at −80 °C. The pellets were resuspended in 50 µL RIPA buffer consisting of 50 mM Tris, 150 mM NaCl, 1% desoxycholic acid sodium salt, 1% Triton X-100, 1 mM EDTA and 0.1% sodium dodecyl sulfate (SDS, pH 7.49) with a protease (cOmplete™-ULTRA-Mini-Tablets, Roche Austria, Vienna, Austria) and phosphatase inhibitor (PhosSTOP™ Easypack, Roche Austria, Vienna, Austria). 30 mg of the native epithelium were homogenized in 300 µL RIPA buffer using a mixer mill as described above. Determination of protein concentration, SDS–polyacrylamide gel electrophoresis, blotting and detection of proteins were performed as described before [53] using 20 µg protein/well (eqJE) or 30 µg protein/well (eqCE). The signal was visualized with Clarity™ Western ECL Substrate (Bio-Rad Laboratories, Vienna, Austria) using a UVP ChemStudio Series Western Blot Imager with the Vision Works^®^ Analysis Software (Analytik Jena GmbH, Jena, Germany) and analysed with Fiji ImageJ64 [52]. β-ACTIN was used as loading control for normalization of each semi-quantitative blot. At least two technical replicates were performed for each sample and target protein. If necessary, the membranes were stripped with a Na-citrate buffer (pH 2.2) and subsequently the protocol was repeated for other target proteins. Antibodies used for Western blotting are listed in Additional file 2.

### Statistics

Statistical analyses were performed using Sigma Plot 14.5 (Systat Software GmbH, Frankfurt, Germany). If not described otherwise, results are shown as boxplots, where boxes represent the median ± 25th and 75th percentiles (box), 10th and 90th percentiles (whiskers). Technical replicates (n) were pooled for each donor animal (i.e., biological replicate, N) for statistical analysis. The data were checked for normality and equal variance using a Shapiro–Wilk and Brown-Forsythe test, respectively. Heatmaps and scatter plots were created using GraphPad Prism 10.1.0 (GraphPad Software, Boston, MA, USA). Enteroids cultivated with different media compositions were compared to the native epithelium of the same donor animal using a Mann–Whitney Rank Sum Test. For determination of the growth rate, enteroids cultivated with DM1-4 were compared to those cultivated with PM, also using a Mann–Whitney Rank Sum Test. Significance was assumed at *p* < 0.05. One asterisk indicates (*) *p* < 0.05, two asterisks (**) *p* < 0.01 and three asterisks (***) *p* < 0.001.

## Results

The aim of this study was to identify culture conditions for eqJE and eqCE that propagate their differentiation to mimic the in vivo intestinal epithelium and thus to create an optimal in vitro model for investigating the pathophysiology of equine intestinal disorders. We compared enteroids cultivated in PM and four differently composed DM to the native epithelium of the same donor horses. The enteroids were characterized morphologically and histologically as well as on gene and protein expression level. In the following, the results are shown first for eqJE and then for eqCE.

### Morphology of eqJE

**Figure 2:**
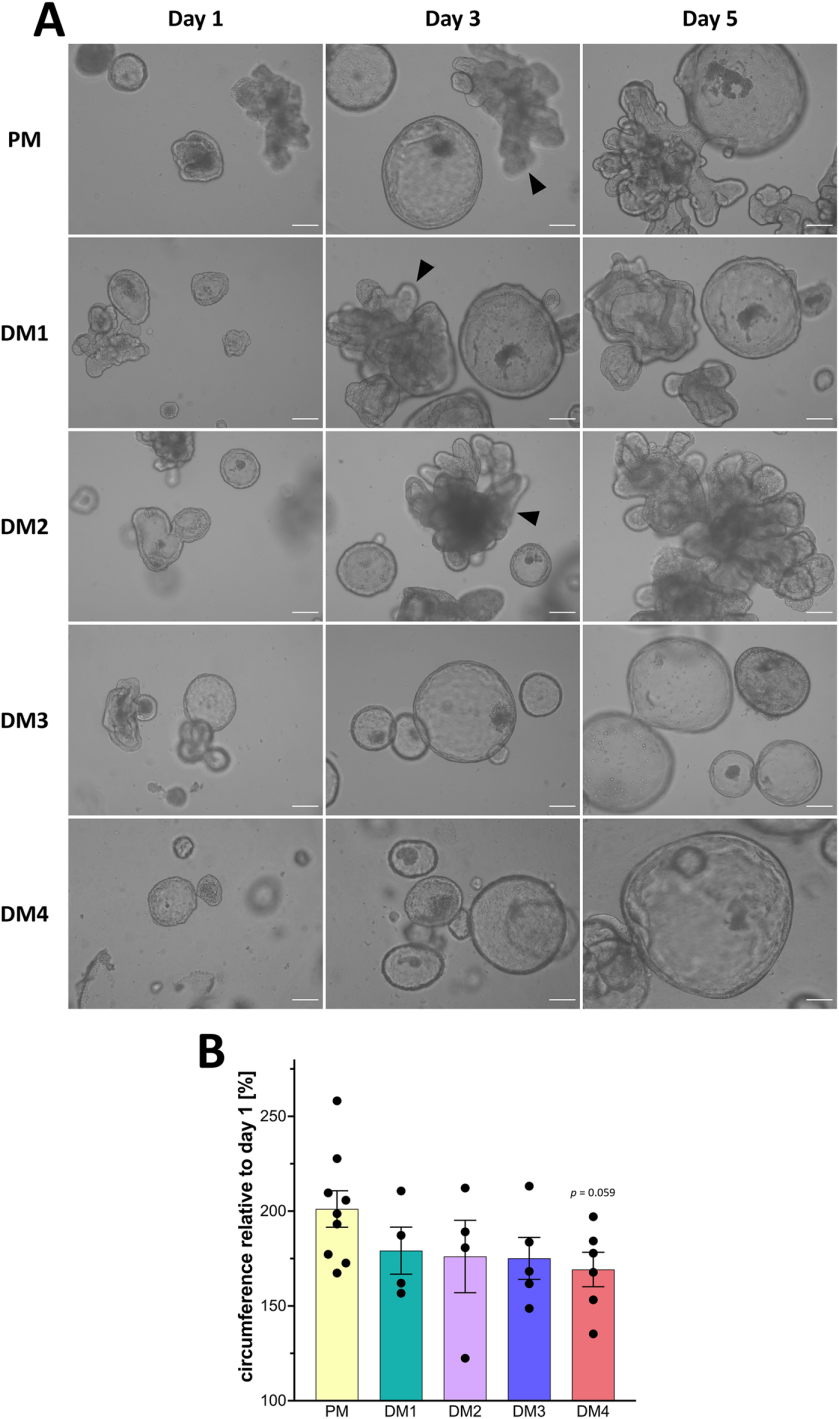
**Morphological characterization of eqJE.** **A** Representative brightfield images of eqJE cultivated with differently composed media on day 1, day 3 and day 5 after splitting. Enteroids cultivated with PM, DM1 and DM2 showed both spherical growth and budding (black arrowheads), whereas in DM3 and DM4 the growth was mainly spherical. Scale bars: 100 µm. **B** Growth rates of eqJE cultivated with differently composed media after four days of cultivation. Means ± SEM; Mann–Whitney Rank Sum Test versus PM; Dots represent biological replicates; *N* = 9 (PM)/4 (DM1, DM2)/5 (DM3)/6 (DM4).

As shown in Figure 2A, eqJE cultivated in PM, DM1 and DM2 showed both spherical growth and budding behavior, whereas in DM3 and DM4 they appeared mainly spherical. With increasing cultivation time, the lumen of the eqJE became dark, possibly due to shedding of cell debris and mucus into the lumen. After approximately 6 days (DM1 and DM2) and 5 days (DM4) in culture, some enteroids appeared less precisely defined, suggesting a loss of epithelial integrity, whereas in PM and DM3 eqJE maintained an intact epithelial morphology.

The growth rate was determined by measuring the circumference of the enteroids at the beginning and after four days of cultivation. The relative growth was calculated and revealed that eqJE cultivated in PM approximately doubled their size within four days. No statistical differences compared to any of the DM were observed. However, enteroids in DM4 showed a tendency towards reduced growth compared to those in PM (*p* = 0.059, Figure 2B).

### Histological evaluation of eqJE

**Figure 3:**
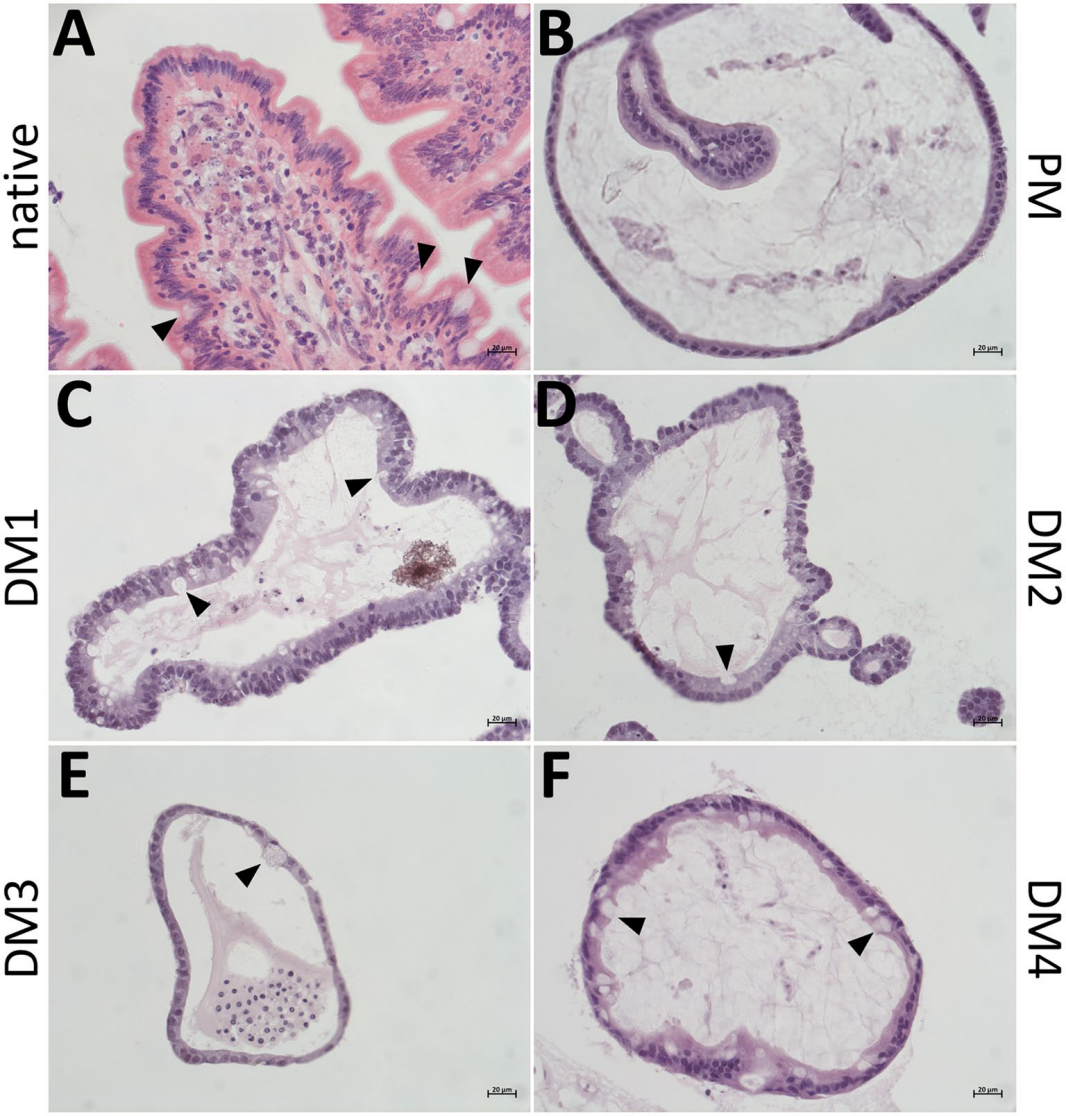
**Representative images of H&E stained cryosections of the native jejunum epithelium (A) in comparison with eqJE cultivated with differently composed media (B–F).** Goblet cells could be observed in the native tissue (**A)** and eqJE cultivated in DM1 (**C)**, DM2 (**D)**, DM3 (**E)** and DM4 (**F)** (black arrowheads). Scale bars: 20 µm.

H&E stainings of native jejunum epithelium and eqJE cultivated with the different media demonstrated that all enteroids formed an intact single layered epithelium (Figure 3). Cell debris and mucus were observed in the lumen of the enteroids. Mucus producing goblet cells were present in the native jejunum epithelium (Figure 3A, Additional file 3A) as well as in all eqJE (Figures 3C–F, Additional file 3C–F), except for enteroids cultivated in PM (Figure 3B, Additional file 3B).

**Figure 4:**
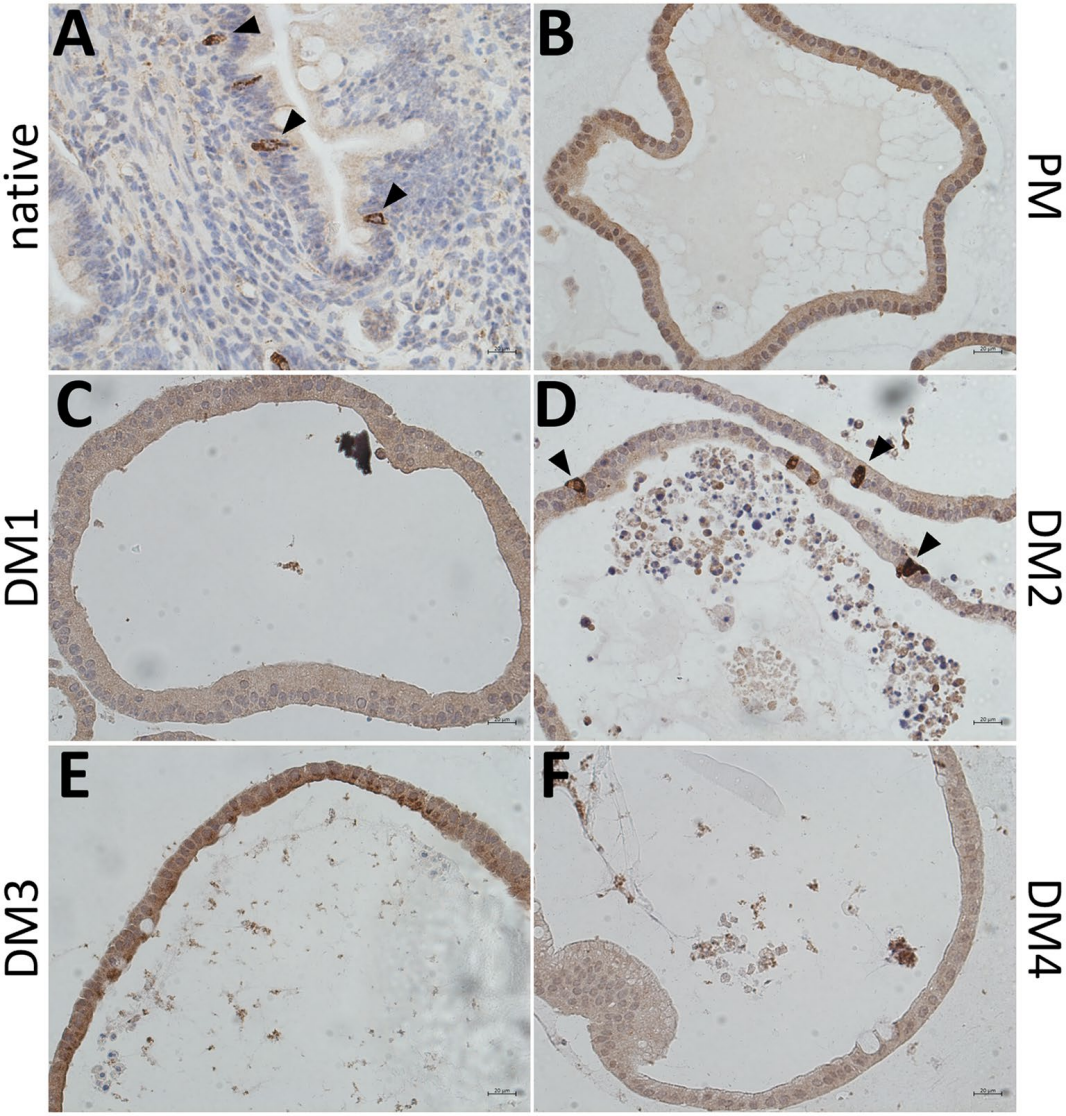
**IHC staining of the enteroendocrine cell marker CHGA in the native jejunum epithelium (A) and in eqJE cultivated with differently composed media (B**–**F).** Black arrowheads indicate CHGA positive cells, which could be observed in the native jejunum epithelium (**A)** as well as in eqJE cultivated in DM2 (**D)**. Scale bars: 20 µm.

IHC stainings of the enteroendocrine cell marker CHGA revealed CHGA positive cells in the native jejunum epithelium (Figure 4A) as well as in eqJE cultivated with DM2 (Figure 4D), whereas no CHGA positive cells could be observed in eqJE cultivated with PM, DM1, DM3 and DM4 (Figures 4B, C, E, F).

### mRNA expression in eqJE

**Figure 5:**
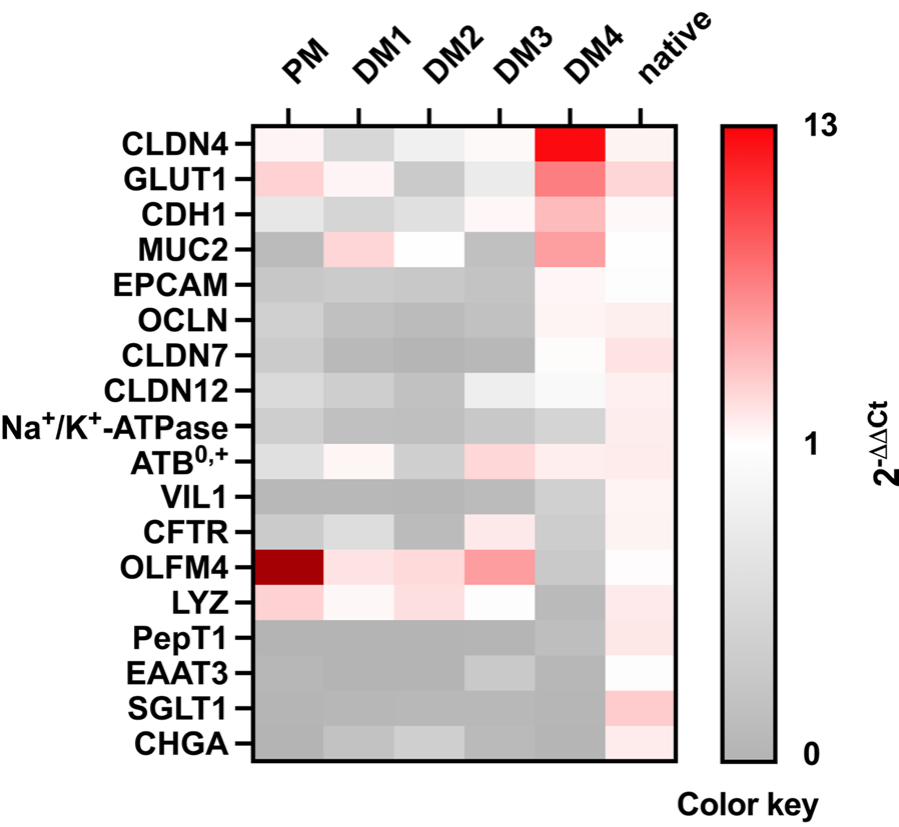
**Heatmap illustrating the mRNA expression levels in eqJE relative to the native jejunum epithelium.**

**Figure 6:**
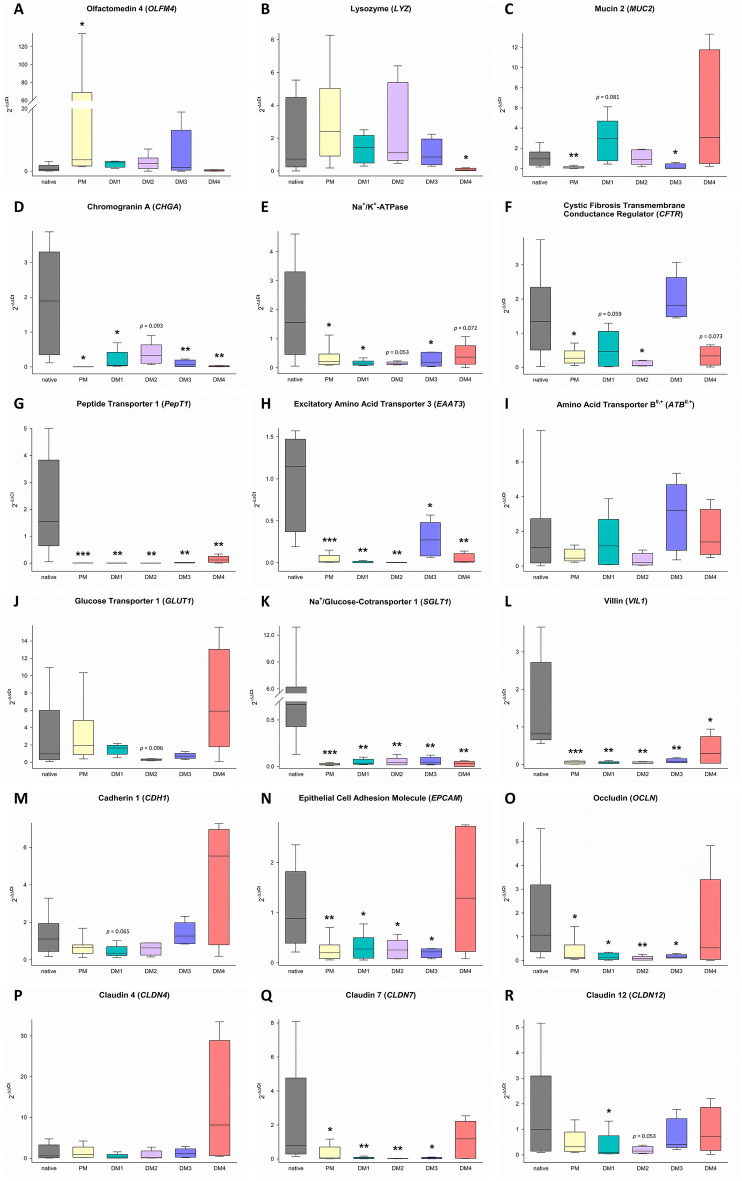
**mRNA expression of marker genes for stem cells (A), Paneth cells (B), goblet cells (C), enteroendocrine cells (D) and enterocytes (E–R) in eqJE compared to the native epithelium.** Mann–Whitney Rank Sum Test versus native jejunum epithelium; *p < 0.05, **p < 0.01, ***p < 0.001; N ≥ 7 (native)/ ≥ 8 (PM)/ ≥ 5 (DM1, DM2) / ≥ 4 (DM3, DM4).

To evaluate the level of differentiation of different epithelial cell types in eqJE, we assessed the gene expression levels of specific cell markers relative to that in the native epithelium of the same donor horses (Figures 5 and 6). The expression of the ISC marker *OLFM4* was significantly higher in enteroids cultivated in PM (Figure 6A), whereas the expression of the Paneth cell marker *LYZ* was significantly lower in eqJE cultivated in DM4 compared to the native jejunum epithelium (Figure 6B). *MUC2*, a marker for mucus producing goblet cells, was expressed significantly less in enteroids cultivated in PM and DM3 compared to the native epithelium (Figure 6C). Hormone secreting enteroendocrine cells were assessed via *CHGA*, whose expression was significantly lower in all eqJE compared to the native jejunum epithelium, except for those cultivated in DM2 (Figure 6D).

Regarding the enterocyte markers (Figures 6E–R), *SGLT1*, *PepT1* and *EAAT3* were expressed significantly less, whereas *ATB0*, *GLUT1*, *CDH1* and *CLDN4* showed no difference in all eqJE compared to the native epithelium. mRNA levels of *Na*^+^*/K*^+^*-ATPase* in eqJE cultivated in PM, DM1 and DM3, of *CFTR* in eqJE in PM and DM2 and of *CLDN12* in eqJE in DM1 were significantly lower compared to the native jejunum epithelium. Expression levels of *EPCAM*, *OCLN* and *CLDN7* were significantly lower in eqJE in all media, except for enteroids in DM4, whose expression level was similar to that in the native jejunum epithelium.

### Protein expression of eqJE

**Figure 7:**
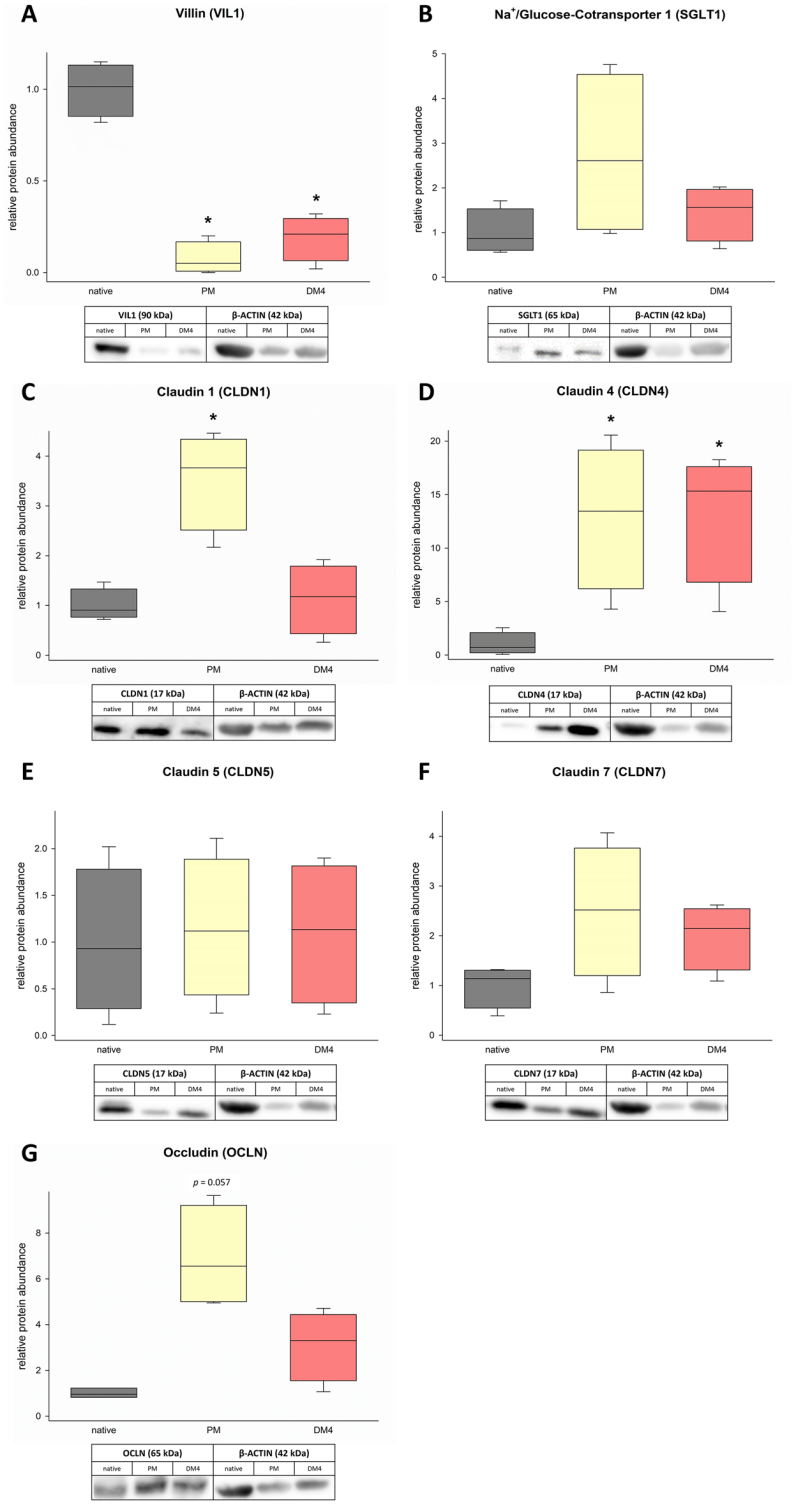
**Protein abundance of the enterocyte markers VIL1 (A), SGLT1 (B), CLDN-1, -4, -5, -7 (C–F) and OCLN (G) in eqJE cultivated in PM and DM4 in comparison to the native jejunum epithelium**. Values were normalized to β-ACTIN and are given relative to the native tissue of the same horses. Mann–Whitney Rank Sum Test versus native tissue; *N* ≥ 3.

Based on the previous observations on mRNA level, indicating the highest similarity of eqJE in DM4 to the native jejunum epithelium, some enterocyte markers were further evaluated on the protein level by Western blot (Figure 7). We detected a significantly lower expression of VIL1 in eqJE cultivated with PM and DM4 compared to the native epithelium (Figure 7A). SGLT1, OCLN, CLDN-5 and -7 were not different in eqJE compared to the native epithelium, irrespective of the culture conditions (Figures 7B, E–G). The abundance of CLDN1 was significantly higher in eqJE in PM and of CLDN4 in eqJE in both PM and DM4 compared to the native epithelium (Figures 7C, D).

### Morphology of eqCE

**Figure 8:**
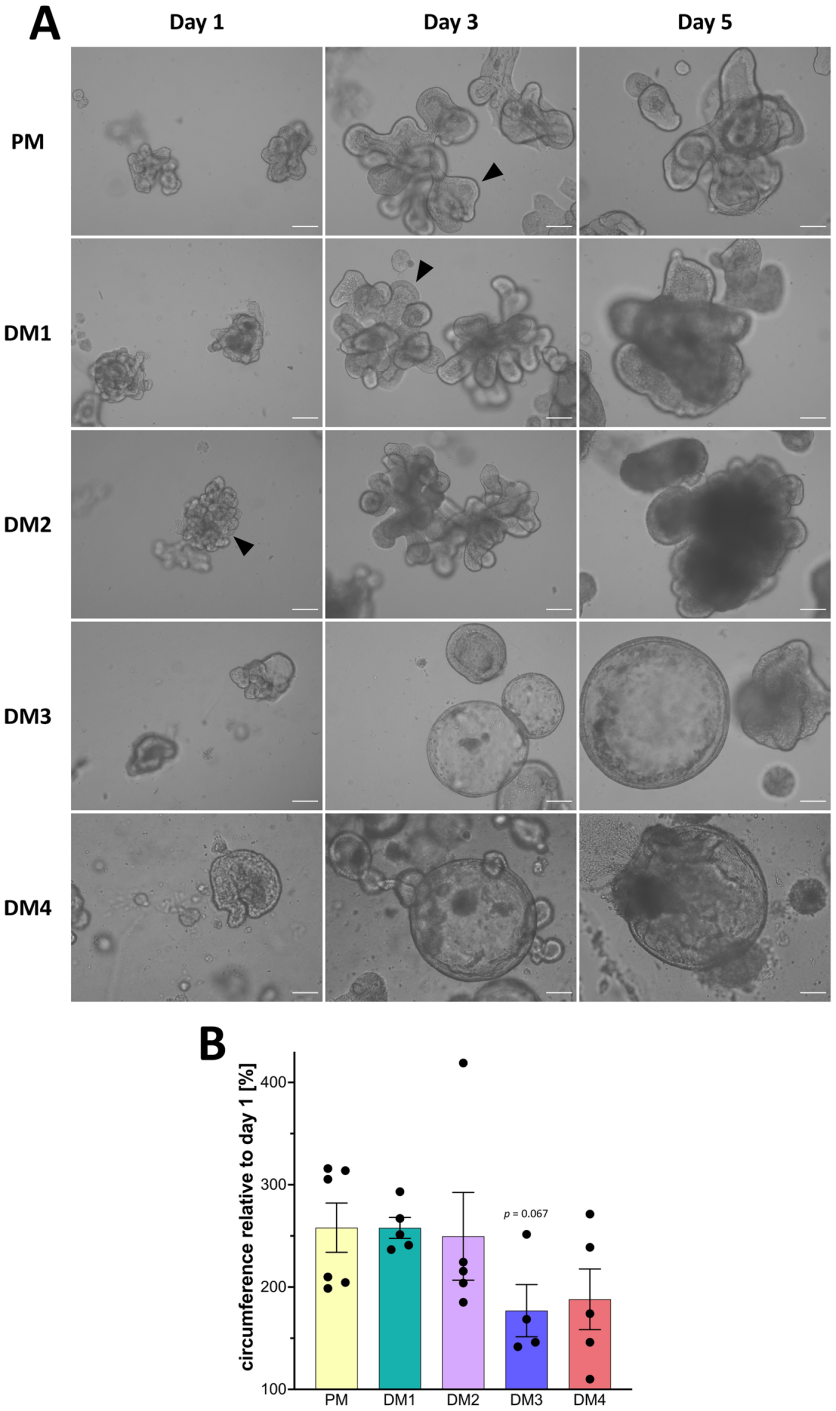
**Morphological characterization of eqCE.** **A** Representative brightfield images of eqCE cultivated with differently composed media at day 1, day 3 and day 5 after splitting. Spherical growth and extensive budding (black arrowheads) were observed in PM and DM1 and DM2, whereas in DM3 and DM4 the growth was mainly spherical. Scale bars: 100 µm. **B** Growth rates of eqCE cultivated with differently composed media after 4 days of cultivation. Means ± SEM; Mann–Whitney Rank Sum Test versus PM; Dots represent biological replicates; *N* = 6 (PM)/5 (DM1, DM2, DM4)/4 (DM3).

As shown in Figure 8A, eqCE also developed various morphologies under the different culture conditions. Similar to eqJE, eqCE cultivated with PM, DM1 and DM2 showed both spherical growth and extensive budding, whereas with DM3 and DM4 they appeared mainly spherical. With increasing cultivation time, a darker lumen could also be observed in eqCE, indicating accumulation of cell debris and mucus. Especially in DM3 (day 6) and DM4 (day 5), the eqCE developed a destructed and deformed epithelial lining after prolonged cultivation.

To further evaluate the morphological differences, the growth rate of the eqCE was determined (Figure 8B). After 4 days of cultivation, eqCE cultivated with PM, DM1 and DM2 reached a circumference of approximately 150% of their initial size. No differences were observed in any of the groups compared to PM. However, enteroids in DM3 showed a tendency towards reduced growth (*p* = 0.067, Mann–Whitney Rank Sum Test).

### Histological evaluation of eqCE

**Figure 9:**
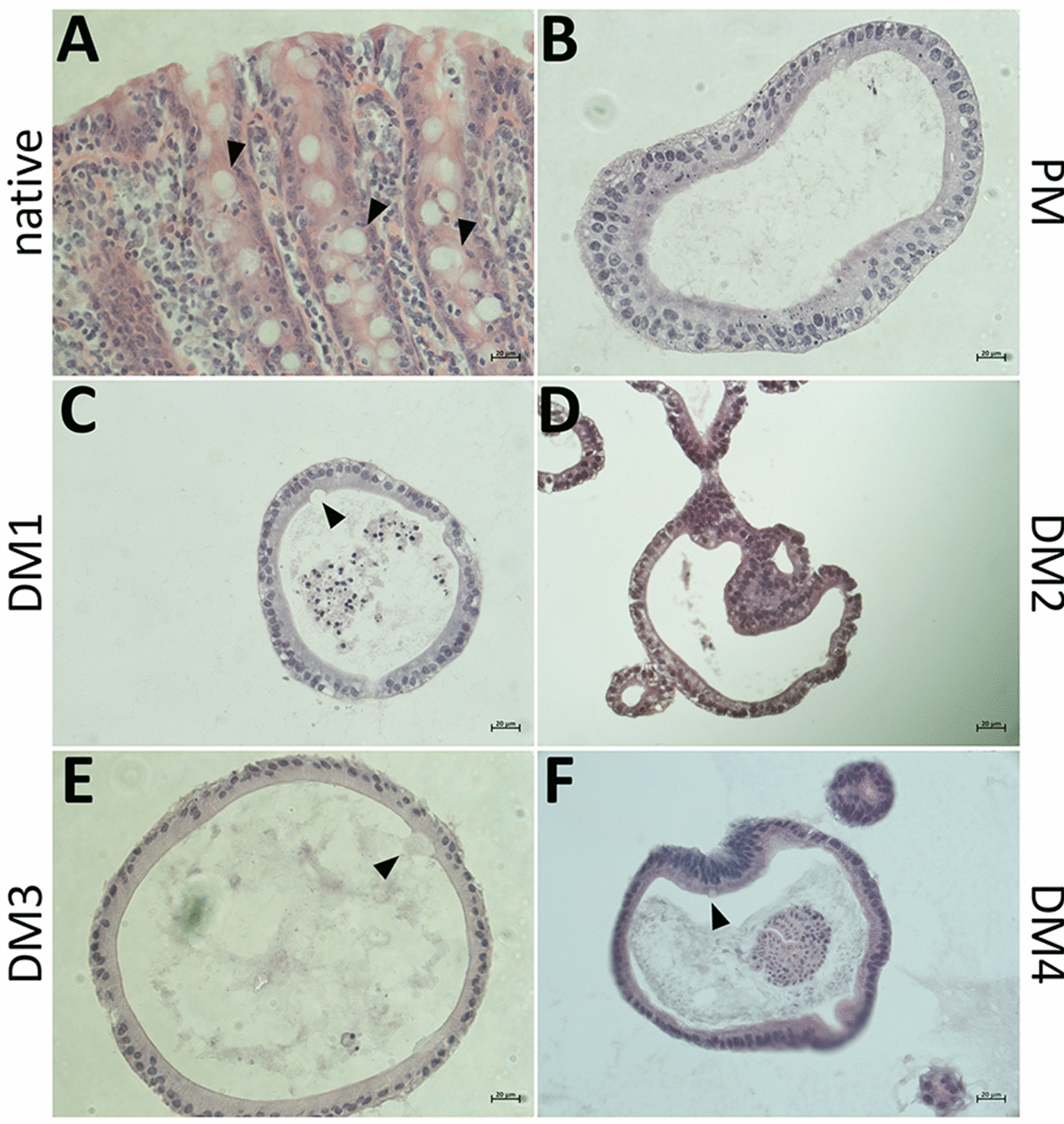
**Representative images of H&E stained cryosections of the native colon epithelium (A) in comparison to eqCE cultivated with differently composed media (B–F).** Goblet cells are marked with black arrowheads and could be observed in the native tissue (**A)** and eqCE cultivated in DM1 (**C)**, DM3 (**E)** and DM4 (**F)**. Scale bars: 20 µm.

H&E stainings of equine colon epithelium and eqCE cultivated with the different media compositions were performed (Figure 9). All enteroids formed an intact single layered epithelium and the lumen of the enteroids was partly filled with cell debris and mucus. Mucus producing goblet cells were observed in the native epithelium (Figure 9A, Additional file 4A) and in eqCE cultivated with DM1, DM3 and DM4 (Figures 9C, E, F, Additional files 4C, E and F). No goblet cells could be observed in eqCE cultivated with PM and DM2 (Figures 9B, D, Additional files 4B, D).

**Figure 10:**
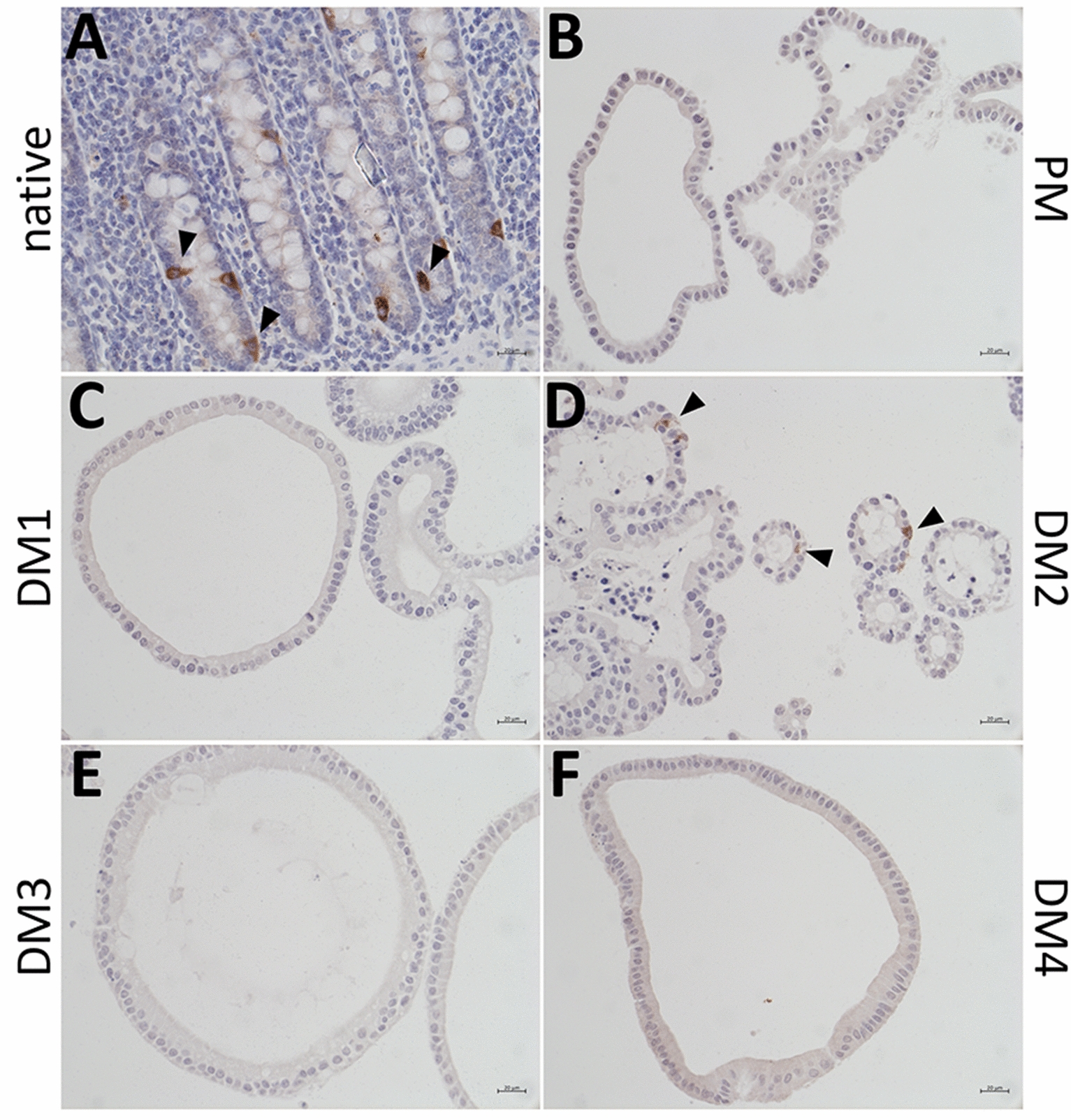
**IHC staining of the enteroendocrine cell marker CHGA in the native colon epithelium (A) in comparison with eqCE cultivated with differently composed media (B–F).** CHGA positive cells could be observed in the native tissue (**A)** and in eqCE cultivated in DM2 (**D)** (black arrowheads). Scale bars: 20 µm.

IHC staining revealed CHGA positive cells in the native colon epithelium (Figure 10A) and in enteroids cultivated with DM2 (Figure 10D). No CHGA positive cells could be observed in eqCE cultivated with PM, DM1, DM3 and DM4 (Figures 10B, C, E and F).

### mRNA expression of eqCE

**Figure 11:**
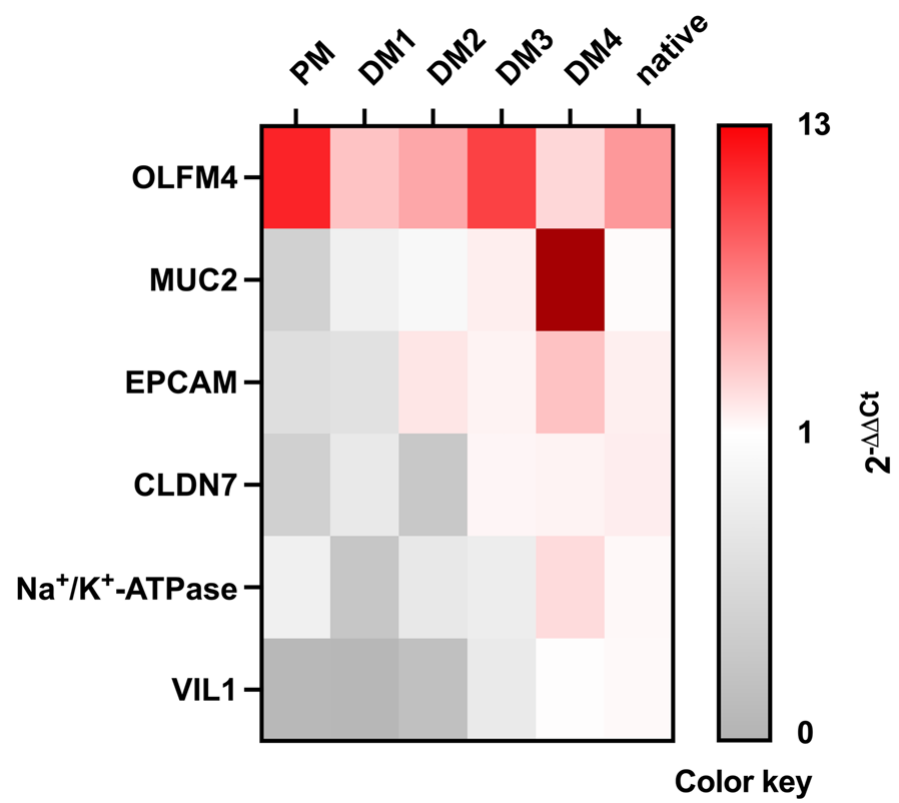
**Heatmap showing mRNA expression levels in eqCE relative to the native colon epithelium.**

**Figure 12:**
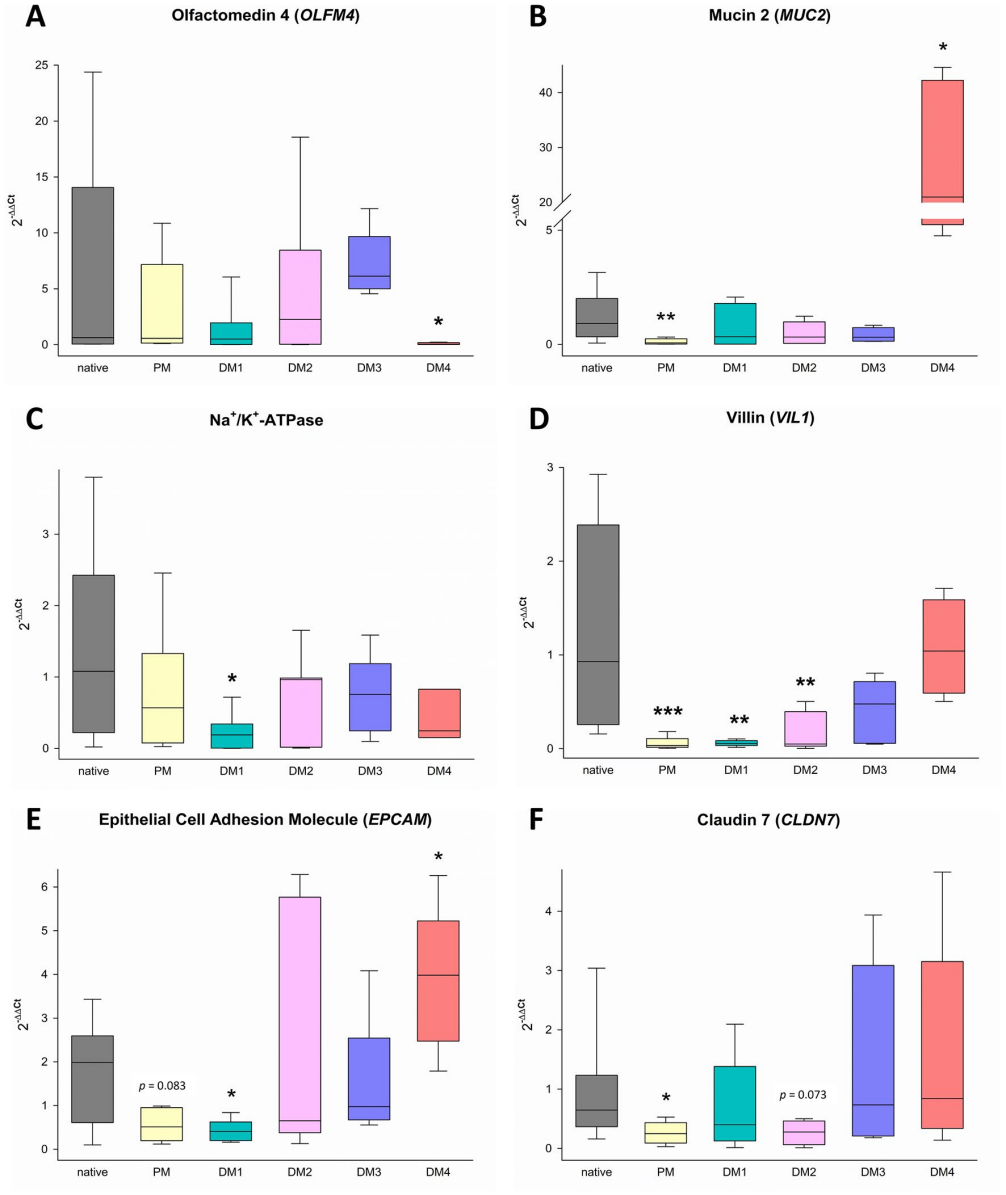
**mRNA expression of specific cell markers for stem cells (A), goblet cells (B) and enterocytes (C–F) in eqCE in comparison to the native epithelium**. Mann–Whitney Rank Sum Test versus native colon epithelium; *p < 0.05, **p < 0.01, ***p < 0.001; N ≥ 7 (native, PM)/ ≥ 6 (DM1)/ ≥ 5 (DM1, DM3)/ ≥ 4 (DM4).

We assessed the mRNA expression of markers for ISC, goblet cells and enterocytes in eqCE cultivated under different conditions. As shown in Figures 11 and 12, the expression of *OLFM4* was significantly lower in eqCE cultivated with DM4 (Figure 12A), whereas *MUC2* and *EPCAM* showed a significantly higher expression in eqCE in DM4 compared to the native colon epithelium (Figures 12B, E). mRNA levels of *MUC2*, *VIL1* and *CLDN7* were significantly lower in eqCE cultivated with PM compared to the native colon epithelium (Figures 12B, D and F). The expression of *Na*^+^*/K*^+^*-ATPase*, *VIL1* and *EPCAM* was significantly lower in eqCE cultivated with DM1 and that of *VIL1* in eqCE in DM1 and DM2 compared to the native epithelium (Figures 12C–E).

### Protein expression of eqCE

**Figure 13:**
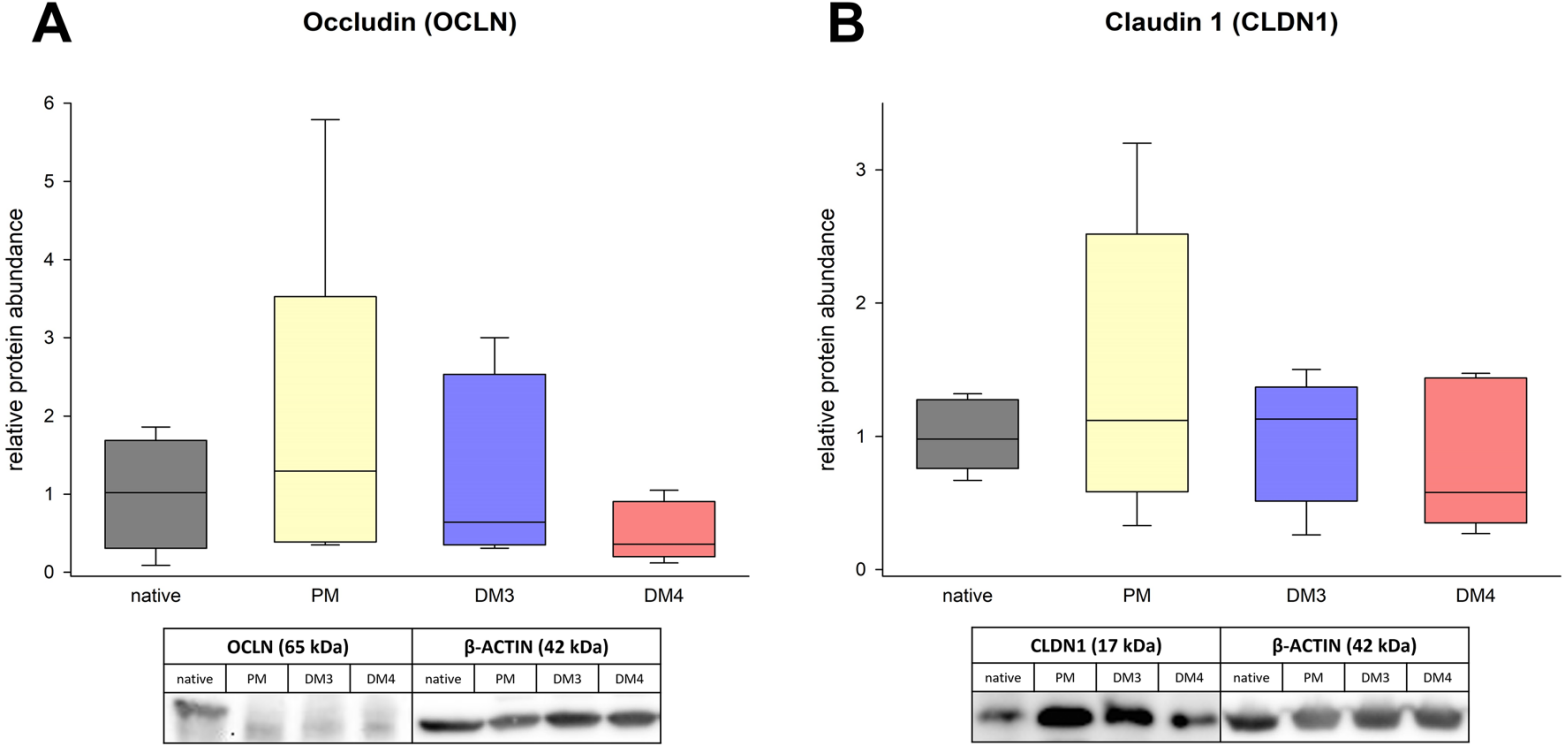
**Protein abundance of OCLN (A) and CLDN1 (B) in eqCE cultivated in PM, DM3 and DM4 in comparison to the native epithelium.** Values are normalized to β-ACTIN and given relative to the mean of the native epithelium of the same horses. Mann–Whitney Rank Sum Test versus native tissue; *N* = 6 (native, PM)/5 (DM3, DM4).

The enterocyte differentiation in eqCE was also assessed on protein level using Western blot (Figure 13). Since in eqCE both DM3 and DM4 showed a tendency to promote the differentiation of enterocytes on mRNA level, these analyses were performed using eqCE cultivated in PM as well as DM3 and DM4. There was no difference in the expression of OCLN (Figure 13A) and CLDN1 (Figure 13B) in eqCE compared to the native colon epithelium, irrespective of the culture conditions.

## Discussion

Three-dimensional enteroids derived from adult ISC are a promising tool for studying pathophysiological mechanisms in the intestinal epithelium with high translational potential to the in vivo situation.

Increased use of these three-dimensional enteroids as an alternative to in vivo experiments will lead to fewer experiments with the respective species, but at the same time to an increased production of the indispensable extracellular matrix, which is often obtained from mouse tumors. This aspect should be assessed as a critical point, particularly with regard to the supposed ethical superiority of in vitro models.

While enteroids may be easily cultivated and propagated under various conditions, induction of a differentiated phenotype resembling the in vivo situation and thus presenting a viable model for translational research is key to use them as a real alternative to in vivo experiments. Additionally, optimal culture conditions for enteroids have been demonstrated to be species specific [26, 29] and commercial cultivation media that are currently available might not fulfill the individual requirements of each species. Equine jejunum enteroids cultivated in a medium supporting the stem cell niche have previously been characterized qualitatively [44, 45]. Stieler Stewart et al. [44] used immunofluorescent stainings and conventional PCR to detect different cell markers in newly isolated enteroids. The stability of cell lineage marker gene expression over six passages was shown by Hellman [45], but with only one biological replicate. Quantitative analyses in direct comparison with the native tissue considering inter-individual variations are missing so far. To the best of our knowledge, no study has been conducted characterizing equine colon enteroids. We aimed to establish culture conditions for the generation of differentiated equine enteroids mimicking the cellular composition and particularly the differentiation grade of enterocytes of the respective epithelium in vivo to study the pathophysiology of equine intestinal disease. Our results emphasize the importance of choosing the ideal culture conditions that appear to be not only species specific but may also differ between intestinal segments.

Usually, enteroids are cultivated in the presence of wingless-related integration site (Wnt), roof plate-specific spondin (R-spondin) and the bone morphogenic protein inhibitor Noggin, which play a crucial role in keeping ISC active [16, 18, 20, 56, 57]. Accordingly, eqJE and eqCE that were cultivated with PM had high expression levels of *OLFM4*, a marker for ISC [56, 58] and a target gene of Notch and Wnt [20], indicating high stem cell activity and proliferation rate in these enteroids. In contrast, with increasing differentiation of the enteroids, *OLFM4* expression decreased and was even significantly lower compared to the native colon epithelium in eqCE cultivated with DM4. At the same time, eqJE showed a low expression of the Paneth cell marker *LYZ* when incubated with DM4. Paneth cells are highly specialized epithelial cells residing at the base of the small intestinal crypts. They secrete antimicrobial peptides and proteins and play a role in the maintenance and modulation of the epithelial stem and progenitor cells [59]. Paneth cells are not found in all animals [60], but their existence has been proven in the equine small intestine as well as in equine jejunum enteroids [44, 45]. Stieler Stewart et al. [44] demonstrated LYZ-positive cells in equine jejunum enteroids using immunofluorescent stainings. A decreased expression of the crypt cell markers *OLFM4* and *LYZ* upon differentiation could also be observed in human enteroids and human enteroid derived 2D monolayers [28, 61].

These results suggest that enterocyte differentiation requires reduction or complete withdrawal of the stem cell niche pathway components Wnt, R-spondin and Noggin and give a first clue that differentiation of the epithelial cells was promoted with increasing impact by cultivation in DM1-4. This is further supported by the mRNA expression levels of enterocyte markers that increasingly resemble the expression levels in the native epithelium. In addition to a low expression of crypt cell markers, Kip et al. [28] and de Lange et al. [61] also showed that the expression of enterocyte markers was increased in human small intestinal enteroids cultivated in media containing less stem cell promoting factors. The expression of sucrase isomaltase, a marker of absorptive enterocytes, was confirmed in equine jejunum enteroids by conventional PCR [44]. As vectorial transepithelial transport is a key function of the intestinal epithelium, we focused on the expression of membrane transporters such as *Na*^+^*/K*^+^*-ATPase*, *CFTR*, *ATB0* and *GLUT1,* which suggested that cultivation of eqJE in DM4 and of eqCE in DM3 supports differentiation of enterocytes similar to the native epithelium in vivo. However, expression levels of the brush border membrane marker *VIL1* as well as the nutrient transporters *PepT1*, *EAAT3* and *SGLT1* were low in all eqJE irrespective of the medium composition. In contrast to that, a study in porcine jejunum enteroids showed no difference in the expression of *SGLT1* and even a higher expression of *CFTR* compared to native epithelium after one week of cultivation in growth medium including stem cell niche supporting components [42]. Hence, there might be species-specific differences in epithelial functions [29] and growth and differentiation requirements.

Nevertheless, the protein abundance of SGLT1 was similar to the native epithelium in eqJE cultivated with both PM and DM4. This phenomenon could be observed for most proteins we assessed, demonstrating that mRNA and protein expression do not necessarily correspond. In a study with specific cell type enriched small intestinal murine enteroids incongruencies between mRNA and protein levels were shown as well [62]. In our study, there might be a confounding effect by the nature of the samples used, as the isolated epithelium used as native control tissue still contained remnants of the submucosa, despite careful preparation, while enteroids are composed of pure epithelial cells. It is an ongoing discussion whether gene expression reflects biological relevance. Still, qPCR is a good method to compare many different markers as an overview and the abundance of marker genes supports an increasing differentiation of enterocytes in our study. While we compared the enteroids to the intact intestinal epithelium in our study, it might also have been an interesting approach to isolate epithelial crypts and villi separately to get a better impression of the resemblance of the enteroids to the different compartments. Yet, in a pathophysiological situation it is also the whole epithelium with ISC and all more or less differentiated cell types that is confronted with the disease and may contribute to epithelial adaptation or healing.

Adherent intercellular structures such as adherens junctions and tight junctions are critical for maintaining the intestinal barrier [63]. Therefore, we also assessed the expression of proteins from this subgroup to evaluate the degree of differentiation of the enteroids. The gene expression levels of the tested markers showed a close resemblance of eqJE cultivated in DM4 to the native epithelium. Pearce et al. [62] showed that CLDN7 was present in enterocyte rich small intestinal murine enteroids, which is in line with our findings that *CLDN7* was expressed significantly less in all media, except in eqJE cultivated with DM4 and eqCE cultivated with DM3 and DM4, respectively. Likewise, the gene and protein levels of the enterocyte markers *Na*^+^*/K*^+^*-ATPase, VIL1* and *CLDN7* in eqCE cultivated with DM3 and DM4 were similar to the native epithelium, whereas mRNA levels of *EPCAM* were even higher in eqCE cultivated with DM4. In contrast, Hellman [45] previously reported a sustained expression of *EPCAM* in equine jejunum enteroids over six passages and no difference in the expression levels between three-dimensional enteroids and enteroid-derived monolayers, despite a general trend towards an increased differentiation of the enterocytes in the monolayer.

Beyond the gene and protein expression level, the spherical morphology of enteroids cultivated with DM3 and DM4 might be considered an indicator for a more differentiated phenotype in these enteroids compared to the multilobular appearance of enteroids in PM but also DM1 and DM2. Enteroids consist of budding structures with a central pseudolumen and surrounding crypt-like domains [16], which are believed to reflect the crypt-villus like regions of the original epithelium. Our results are in line with previous studies reporting a cystic phenotype in differentiated human and murine small intestinal enteroids cultivated in media free of Wnt and any Wnt-activators [28, 62], which is comparable to DM3 and DM4 in our study. In contrast, Zou et al. observed [63] increased budding behavior in differentiated human small intestinal enteroids that were also cultivated in a Wnt-free medium. Hence, other factors, such as cultivation time, might also influence the morphological appearance of the enteroids. In porcine jejunal enteroids a trend towards a cystic phenotype and at the same time an increased expression of secretory cell lineages was observed with increasing cultivation time [43]. A reduced proliferation rate was confirmed by circumference measurements, which revealed a tendency towards reduced growth in eqJE cultivated in DM4 and eqCE cultivated with DM3 and DM4. Additionally, enteroids in DM3 and DM4 appeared to have a less defined contour from day 5 onwards. This may be indicative of a high proportion of differentiated cells and a lack of proliferating cells, suggesting a generation time of about 5 days, which is identical to the cell turnover observed in the intestinal epithelium in vivo [6, 14, 44, 64, 65].

Apart from enterocytes of varying differentiation grade, histological stainings confirmed the presence of several cell types of the epithelial lineage, such as goblet cells and enteroendocrine cells. Mucus producing goblet cells were present in eqJE cultivated in all DM, but not in PM, and in eqCE cultivated with DM3 and DM4, which reflects our findings on gene expression level and confirms that medium favoring proliferation of stem cells opposes differentiation into all cell types of the intact epithelium [26]. Interestingly, the abundance of goblet cells has been demonstrated by immunofluorescent staining of MUC2 in equine jejunum enteroids cultured in a medium supporting stem cell proliferation before [44], which contrasts our findings. A study in porcine jejunum enteroids revealed a higher amount of goblet cells in comparison with the native epithelium, which was explained by the addition of the γ-secretase inhibitor DAPT [42]. This might also be the reason for the higher *MUC2* expression in eqCE cultivated in DM4 in our study.

The positive IHC staining of the enteroendocrine cell marker CHGA in the native jejunum epithelium and eqJE cultivated in DM2 correlated with the gene expression levels of *CHGA* which were low under all culture conditions, except in eqJE cultivated in DM2. Interestingly, eqCE cultivated with DM2 also showed CHGA positive cells. This may indicate that the enteroendocrine cells in the equine intestinal epithelium respond to the same signal, despite different effects of the DM on enterocyte differentiation in eqJE and eqCE. A previous study showed that simultaneous inhibition of Notch, Wnt and epidermal growth factor receptor signaling is key for the generation of enteroendocrine cells [51]. This is in contrast with our findings and also another study, which demonstrated a differentiation of enteroendocrine cells in the presence of Wnt signaling [66]. Another possibility might be that the glycogen synthase kinase 3 inhibitor CHIR-99021 and/or the transforming growth factor beta (TGF-β) receptor kinase inhibitor LY2157299 triggered the differentiation of enteroendocrine cells in eqJE and eqCE in our study. This coincides with the results of a study in equine jejunum enteroids supplemented with CHIR-99021 and LY2157299 that also demonstrated the existence of enteroendocrine cells [44]. However, a study in small intestinal murine enteroids demonstrated that treatment with TGF-β induces an enrichment of enteroids with enteroendocrine cells [67]. Taken together, differentiation into enteroendocrine cells might be regulated differently in a species-specific manner, whereas there appear to be more similarities for the general differentiation process.

It has been shown that active Wnt signaling promotes the proliferation of human colonic crypt stem cells and that the Wnt signal can be suppressed by bone morphogenic protein [68]. Therefore, withdrawal of Wnt in DM3 and withdrawal of the bone morphogenic protein inhibitor Noggin in DM4 may have been decisive for differentiation of enterocytes in our study. The lack of R-spondin and Noggin in DM4 is the most striking difference between DM3 and DM4. R-spondin, which acts as a Wnt enhancer, is effective in tissues where the Wnt signaling pathway is already activated [69]. Since DM3 contains no exogenous Wnt source, the effect of R-spondin would rely on endogenous Wnt, whose absence could explain the degree of differentiation and the high comparability of eqCE cultivated in DM3 with the native epithelium. A differentiating effect by withdrawal of Wnt alone was also observed in murine colon enteroids [26]. The lower degree of differentiation in eqJE cultivated in DM3 may be due to the interaction of R-spondin with endogenous Wnt provided by Paneth cells [70].

Noggin plays a crucial role in the enterocyte differentiation along the crypt-villus axis [71, 72]. The withdrawal of Noggin, possibly in combination with DAPT, could have a potent effect on the degree of differentiation of enteroids cultivated in DM4. The γ-secretase inhibitor DAPT induces a high degree of differentiation and reduced stem cell activity [21] by blockade of Notch signaling, which has an essential role in regulating epithelial cell fate [20]. Addition of DAPT has been shown to support human enteroid differentiation before [25, 61, 73] and appears to be crucial in differentiating eqJE with DM4 as well. In combination with Wnt and R-spondin withdrawal, it appears to be more effective in eqCE than in eqJE, because eqCE appeared to be rather over-differentiated in DM4, whereas eqJE only approximated the gene expression levels of enterocyte markers in the native epithelium when cultivated in DM4. Another factor might be the withdrawal of the Rho kinase inhibitor Y-27632 dihydrochloride and the glycogen synthase kinase 3 inhibitor CHIR-99021 in DM4, which has previously been shown to induce differentiation in a canine enteroid-derived 2D monolayer [74]. A similar effect of the combined withdrawal of Wnt, R-spondin and Noggin in eqJE was also observed in human colonoids [26].

Taken together, our observations indicate a close resemblance of eqJE and eqCE to the native epithelium. Particularly when focusing on enterocyte differentiation, eqJE cultivated under depletion of any factors activating the stem cell niche and with addition of a Notch signal inhibitor (DM4) and eqCE cultivated without Wnt (DM3) mimic the in vivo conditions best. While most studies so far have impressively shown a differentiated phenotype of enteroids cultivated with modified media in comparison to proliferative conditions, there are only few studies comparing these enteroids to the in vivo tissue they are supposed to mimic. Our study shows that reaching a similar state of differentiation is rather complex and that both species-specific as well as segment-specific requirements may need to be considered in order to achieve this goal. The discrepancy between differently composed DM in our study, e.g., regarding the abundance of enteroendocrine cells, demonstrates that the optimal conditions vary for each cell type in the epithelium. While an exact replication of the in vivo situation will probably never be achieved under artificial culture conditions, it is of utmost importance to at least attempt to characterize the models used to conduct research with the best possible translational potential. In the future, we will use this equine enteroid model to investigate equine gastrointestinal disease and to identify new therapeutic targets.

### Supplementary Information

**Additional file 1: Primers used for qPCR.**

**Additional file 2: Antibodies used for Western blot.**

**Additional file 3: Representative images of PAS stained cryosections of the native jejunum epithelium (A) in comparison with eqJE cultivated with differently composed media (B–F).** Goblet cells could be observed in the native tissue (**A)** and eqJE cultivated with DM1 (**C)**, DM2 (**D)**, DM3 (**E)** and DM4 (**F)**. Scale bars: 20 µm.

**Additional file 4: Representative images of PAS stained cryosections of the native colon epithelium (A) in comparison to eqCE cultivated in differently composed media (B–F).** Goblet cells could be observed in the native tissue (**A)** and eqCE cultivated with DM1 (**C)**, DM3 (**E)** and DM4 (**F)**. Scale bars: 20 µm.

## Data Availability

All data generated or analysed during this study are included in this published article and its supplementary information files. The data that support the findings of this study are available upon reasonable request from the corresponding author.
