# Supplementary material for: A matter of differentiation: equine enteroids as a model for the in vivo intestinal epithelium
Source: Vet Res. 2024 Mar 16;55:30. doi: 10.1186/s13567-024-01283-0 (PMC10943904; doi:10.1186/s13567-024-01283-0)
Supplement: Supplementary file 1 — Additional file 1: Primers used for qPCR. [file 13567_2024_1283_MOESM1_ESM.docx]

Additional file 1: Primers used for qPCR

| **Classification** | **Gene name** | **GenBank**  **accession number** | **Primer sequence**  **(5´‑3´)** | **Annealing temperature**  **[°C]** | **Amplicon length**  **[bp]** |
| --- | --- | --- | --- | --- | --- |
| **Reference genes** | HPRT1 | [XM_023634464.1](https://www.ncbi.nlm.nih.gov/entrez/viewer.fcgi?db=nucleotide&id=1333710417) | F: GGGATTTGAATCACGTTTGTGTC  R: CTCCAGATGTTTCTCCAACTCAACC | 60 | 65 |
|  | PPIA | [XM_001496943.5](https://www.ncbi.nlm.nih.gov/entrez/viewer.fcgi?db=nucleotide&id=1333652603) | F: GCCAAGACTGAGTGGTTGGAT  R: TTGCTGGTCTTGCCATTCCT | 60 | 113 |
|  | RPL4 | [XM_001497094.4](https://www.ncbi.nlm.nih.gov/entrez/viewer.fcgi?db=nucleotide&id=1333550561) | F: CCAGGCCAAGAATCACAAACTC  R: TGCTTTCTTCCCTACCACAGG | 60 | 109 |
|  | RPL32 | [XM_001492042.6](https://www.ncbi.nlm.nih.gov/entrez/viewer.fcgi?db=nucleotide&id=1333580591) | F: TGCAACAAATCGTACTGTGC  R: GGCATTGGGATTGGTGATTCTG | 60 | 111 |
| **Stem cell**  **marker** | OLFM4 | XM_001493449.4 | F: CAGCTCTGTGTCCCAGTCGT  R: GGTGGTGTCTGGCAACGTA | 60 | 91 |
| **Paneth cell marker** | LYZ | XM_001494130.3 | F: CTGTCCAGGGCAAGGTCTTT  R: CTGGCCAAACAGACCCAGTT | 60 | 109 |
| **Goblet cell**  **marker** | MUC2 | XM_023654909.1 | F: GAAGAGACCAATGTGGCTGC  R: GAAGAGACCAATGTGGCTGC | 60 | 101 |
| **Enteroendocrine cell marker** | CHGA | XM_023628306.1 | F: TGAGGTCATCTCTGACACGC  R: CAGGATCCGTTCATCTCCTCG | 60 | 91 |
| **Enterocyte markers** | VIL1 | XM_023642347.1 | F: TCGTGGTGAAGCAGGGATAC  R: CATACGATTTGGCGTTGCTCC | 60 | 90 |
|  | Na^+^/K^+^-ATPase | XM_023640223.1 | F: TCCAGCAGGGGATGAAGAAC  R: CACCCATTCCAGGGCAGTAG | 60 | 93 |
|  | CFTR | NM_001110510.1 | F: CTGGAGCAGGCAAGACTTCA  R: AGGCATGATCCAGGAAAACTGA | 60 | 119 |
|  | PepT1 | XM_014732161.2 | F: AGGTAGTCCTTGCAGTGAGC  R: CATGGACAGAGCCACATGGA | 60 | 95 |
|  | EAAT3 | XM_023627299.1 | F: GTCACTGTCCTGAGTGGGCTT  R: GAATCGGAAGGGGTTCTTTCG | 60 | 90 |
|  | ATB0 | XM_001917363.4 | F: GTTCCTGGATCTTGTGAGGAAT  R: TCACCTGGGTTCCGTTGATG | 60 | 107 |
|  | GLUT1 | [NM_001163971.2](https://www.ncbi.nlm.nih.gov/entrez/viewer.fcgi?db=nucleotide&id=1540583170) | F: TACGTGGAGCAACTCTGTGG  R: AATCTCATCGAAGGTCCGGC | 59 | 120 |
|  | SGLT1 | [NM_001081872.1](https://www.ncbi.nlm.nih.gov/entrez/viewer.fcgi?db=nucleotide&id=126352557) | F: GCTTTGAATGGAACGCCCTG  R: TGGCCTCCAAATCGCTTCTT | 57 | 118 |
|  | CDH1 | XM_023637192.1 | F: TGATTCGCAATGATGTGGCG  R: GCTGCCTTCAGGTTCTCATCA | 60 | 109 |
|  | EPCAM | XM_023619127.1 | F: AGGGTCTACAAGCTGGTTATTT  R: CATCACGCATTGAGTTCCCT | 60 | 104 |
|  | OCLN | XM_023618237.1 | F: GCCCTCTGCAACCAGTTCTA  R: AATGGCAATAGCCTCCTGGG | 60 | 102 |
|  | CLDN4 | XM_005598688.3 | F: TCTTCAGTTTGAGAGCGCCA  R: GAAGCCGCCAAGTGTCTTTC | 60 | 106 |
|  | CLDN7 | XM_001503052.5 | F: CATTTTCATCGTGGCAGGTCTT  R: TATTCATGGGGACCAAGGGGT | 60 | 98 |
|  | CLDN12 | XM_014739003.2 | F: TGTAACACCGCCTTCAGGTC  R: GCCAGGAAAAACAGCAGTCC | 60 | 104 |

F: forward primer, R: reverse primer; bp: base pairs; *HPRT1:* hypoxanthine phosphoribosyltransferase 1, *PPIA:* peptidylprolyl isomerase A, *RPL4:* ribosomal protein L4, *RPL32:* ribosomal protein L32, *OLFM4:* olfactomedin 4, *LYZ:* lysozyme, *MUC2:* mucin 2, *CHGA:* chromogranin A, *VIL1*: villin 1, *Na^+^/K^+^-ATPase (ATP1A1):* sodium/potassium-transporting ATPase subunit alpha-1, *CFTR:* cystic fibrosis transmembrane conductance regulator, *PepT1 (SLC15A1):* peptide transporter 1*, EAAT3:* excitatory amino acid transporter 3, *ATB0:* amino acid transporter B0, *GLUT1:* glucose transporter 1, *SGLT1:* sodium/glucose-cotransporter 1, *CHD1:* cadherin 1, *EPCAM:* epithelial cell adhesion molecule, *OCLN:* occludin, *CLDN4:* claudin 4, *CLDN7:* claudin 7*, CLDN12:* claudin 12.
