# Supplementary material for: A matter of differentiation: equine enteroids as a model for the in vivo intestinal epithelium
Source: Vet Res. 2024 Mar 16;55:30. doi: 10.1186/s13567-024-01283-0 (PMC10943904; doi:10.1186/s13567-024-01283-0)
Supplement: Supplementary file 2 — Additional file 2: Antibodies used for Western blot. [file 13567_2024_1283_MOESM2_ESM.docx]

Additional file 2: Antibodies used for Western blot

| **Target** | **Primary**  **antibody** | **Manufacturer,**  **catalogue number** | **Species specificity** | **Dilution** | **Secondary**  **antibody** | **Manufacturer,**  **catalogue number** | **Dilution** |
| --- | --- | --- | --- | --- | --- | --- | --- |
| VIL1 | Rabbit Anti- Villin  (p-IgG) | Thermo Fisher Scientific™, Vienna, Austria, #PA5-78222 | Human, mouse | 1:200 | Goat-anti-rabbit HRP | Cell Signaling #7074 | 1:5,000 |
| SGLT1 | Rabbit Anti-SGLT1  (p-IgG) | Bioss Antibodies Inc., Woburn, MA, USA; #bs-1128R-TR | Human, mouse, rat | 1:200 |  |  |  |
| OCLN | Rabbit Anti-Occludin (p-IgG) | Invitrogen #40-4700 | Canine, human, mouse, rat | 1:500 |  |  |  |
| CLDN4 | Rabbit Anti- Claudin 4 (p-IgG) | Invitrogen #36-4800 | Mouse, rat | 1:500 |  |  |  |
| CLDN5 | Rabbit Anti- Claudin 5 (p-IgG) | Invitrogen #PA5-99415 | Bovine, human, mouse, non-human primate, porcine, rat | 1:500 |  |  |  |
| CLDN7 | Rabbit Anti- Claudin 7 (p-IgG) | Invitrogen #34-9100 | Canine, human, mouse | 1:500 |  |  |  |
| CLDN1 | Mouse Anti- Claudin-1 (A-9) (m-IgG) | Santa Cruz Biotechnology, Inc., Heidelberg, Germany; #sc-166338 | Mouse, human, rat | 1:200 | Goat-anti-mouse HRP | Millipore/Sigma #AP181P | 1:5,000 |
| β-ACTIN | Mouse-anti-β-Actin (m-IgG) | Millipore/Sigma#A5441 | Sheep, carp, feline, chicken, rat, mouse, rabbit, canine, pig, human, bovine, guinea pig | 1:5,000 |  |  | 1:5,000 |

VIL1: villin, SGLT1: sodium/glucose-cotransporter 1, OCLN: occludin, CLDN4: claudin 4, CLDN5: claudin 5, CLDN7: claudin 7, CLDN1: claudin 1, p: polyclonal antibody, m: monoclonal antibody.
