# Supplementary figures and images for: A matter of differentiation: equine enteroids as a model for the in vivo intestinal epithelium
Source: Vet Res. 2024 Mar 16;55:30. doi: 10.1186/s13567-024-01283-0 (PMC10943904; doi:10.1186/s13567-024-01283-0)

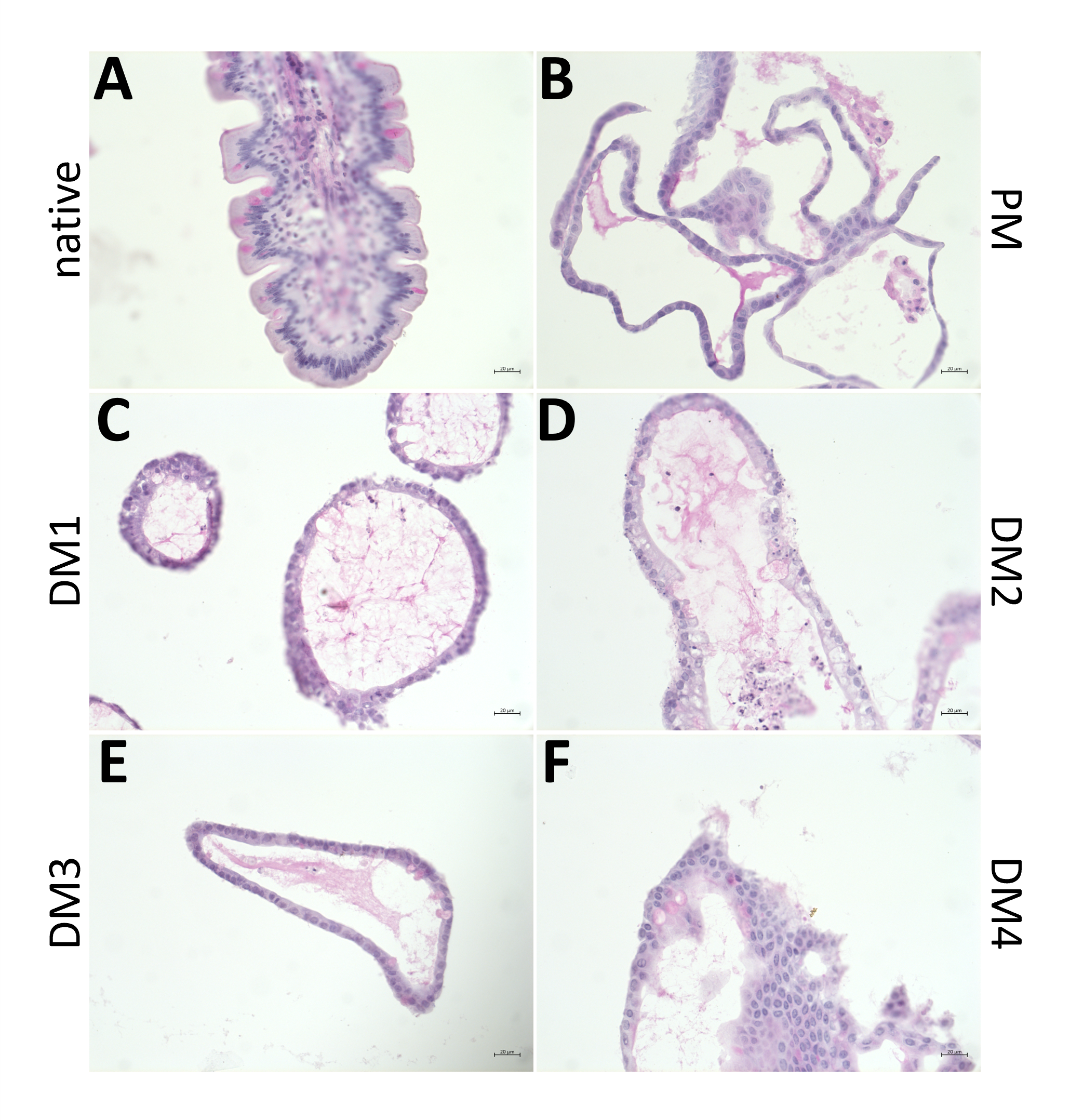

Supplement: Supplementary file 3 — Additional file 3: Representative images of PAS stained cryosections of the native jejunum epithelium (A) in comparison with eqJE cultivated with differently composed media (B–F). Goblet cells could be observed in the native tissue (A) and eqJE cultivated with DM1 (C), DM2 (D), DM3 (E) and DM4 (F). Scale bars: 20 µm. [file 13567_2024_1283_MOESM3_ESM.tif]

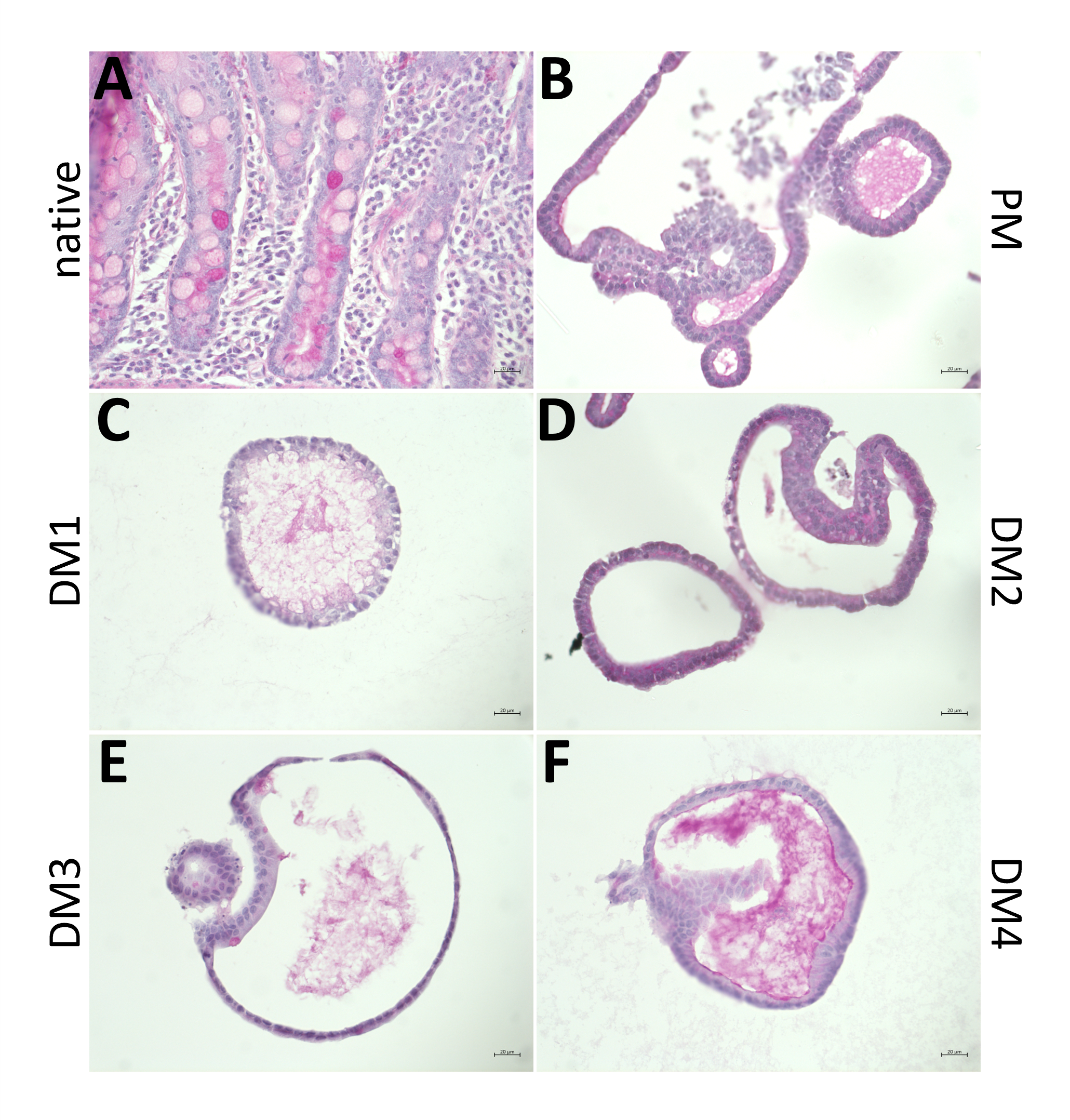

Supplement: Supplementary file 4 — Additional file 4: Representative images of PAS stained cryosections of the native colon epithelium (A) in comparison to eqCE cultivated in differently composed media (B–F). Goblet cells could be observed in the native tissue (A) and eqCE cultivated with DM1 (C), DM3 (E) and DM4 (F). Scale bars: 20 µm. [file 13567_2024_1283_MOESM4_ESM.tif]
